# Th1 cells contribute to retinal ganglion cell loss in glaucoma in a VCAM-1-dependent manner

**DOI:** 10.1186/s12974-024-03035-5

**Published:** 2024-02-05

**Authors:** Chong He, Kun Peng, Xiong Zhu, Zuo Wang, Wenbo Xiu, Gao Zhang, Yang Chen, Chaonan Sun, Xiao Xiao, Donghua Liu, An Li, Yanping Gao, Jinxia Wang, Ping Shuai, Yilian Chen, Ling Yu, Fang Lu

**Affiliations:** 1 Clinical Immunology Translational Medicine Key Laboratory of Sichuan Province, Sichuan Provincial People’s Hospital, University of Electronic Science and Technology of China, Chengdu, China; 2 Yangtze Delta Region Institute (Quzhou), University of Electronic Science and Technology of China, Quzhou, China; 3 Health Management Center, Sichuan Provincial People’s Hospital, University of Electronic Science and Technology of China, Chengdu, China; 4 Department of Ophthalmology, Sichuan Provincial People’s Hospital, University of Electronic Science and Technology of China, Chengdu, China; 5 Department of Ophthalmology, Daping Hospital, Army Medical Center, Army Medical University, Chongqing, China; 6 Department of Prenatal Diagnosis, Chengdu Women’s and Children’s Central Hospital, School of Medicine, University of Electronic Science and Technology of China, Chengdu, China; 7 Department of Clinical Laboratory, Sichuan Cancer Hospital and Institute, Sichuan Cancer Center, School of Medicine, University of Electronic Science and Technology of China, Chengdu, China

**Keywords:** Glaucoma, Retinal neurodegeneration, Retinal ganglion cell, CD4^+^ T cells, VCAM-1, Th1 cells

## Abstract

Glaucoma is a complex neurodegenerative disorder characterized by the progressive loss of retinal ganglion cells (RGC) and optic nerve axons, leading to irreversible visual impairment. Despite its clinical significance, the underlying mechanisms of glaucoma pathogenesis remain poorly understood. In this study, we aimed to unravel the multifaceted nature of glaucoma by investigating the interaction between T cells and retinas. By utilizing clinical samples, murine glaucoma models, and T cell transfer models, we made several key findings. Firstly, we observed that CD4^+^ T cells from glaucoma patients displayed enhanced activation and a bias towards T helper (Th) 1 responses, which correlated with visual impairment. Secondly, we identified the infiltration of Th1 cells into the retina, where they targeted RGC and integrated into the pro-inflammatory glial network, contributing to progressive RGC loss. Thirdly, we discovered that circulating Th1 cells upregulated vascular cell adhesion protein 1 (VCAM-1) on retinal microvessels, facilitating their entry into the neural retina. Lastly, we found that Th1 cells underwent functional reprogramming before reaching the retina, acquiring a phenotype associated with lymphocyte migration and neurodegenerative diseases. Our study provides novel insights into the role of peripheral CD4^+^ T cells in glaucoma pathogenesis, shedding light on the mechanisms underlying their infiltration into the retina and offering potential avenues for innovative therapeutic interventions in this sight-threatening disease.

**Graphical Abstract:**

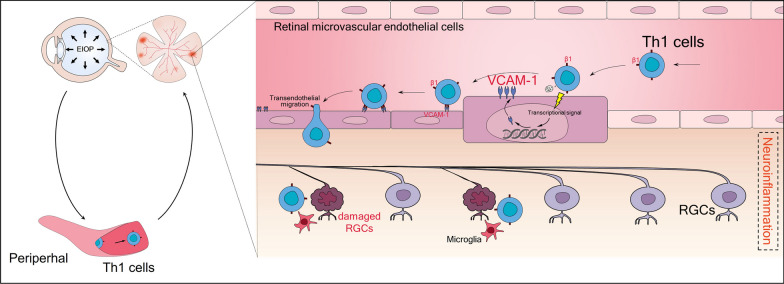

**Supplementary Information:**

The online version contains supplementary material available at 10.1186/s12974-024-03035-5.

## Introduction

Glaucoma, a multifactorial neurodegenerative disorder, leads to a gradual loss of retinal ganglion cells (RGC) and optic nerve (ON) axons, resulting in irreversible visual impairment. This disease remains a significant clinical challenge, primarily due to the limited understanding of its pathogenesis. While elevated intraocular pressure (EIOP) is recognized as the primary risk factor for glaucoma, the existing strategy of reducing IOP proves inadequate in impeding or decelerating disease progression in certain patients [1]. Despite achieving normalization of IOP, the disease often persists and progresses, suggesting the involvement of mechanisms beyond EIOP [2, 3]. Therefore, a comprehensive understanding of the multifaceted nature of glaucoma is imperative for the development of effective interventions capable of attenuating or retarding disease progression.

Neuroinflammation, a common feature of neurodegenerative diseases, including glaucoma, has garnered substantial recognition [4]. Although the retina was conventionally regarded as an immune-privileged organ, recent years have witnessed a shift in this perception [5, 6]. The communication between the retina and the periphery is more dynamic than previously believed, challenging the notion that the retina was isolated from the rest of the body by the blood-retinal barrier (BRB). Activation of the peripheral immune system has emerged as a critical factor in the etiology of glaucoma [7–11]. Our group, alongside others, has provided evidence implicating both innate and adaptive immune components [10, 12–14], with considerable focus on CD4^+^ T cells in the past two decades [12, 15–18]. In experimental glaucoma models, including inducible and genetic mouse models, increased infiltration of CD4^+^ T cells into the retina has been observed. Transfer of CD4^+^ T cells from glaucomatous mice to EIOP-naïve recipients induces RGC loss, while mice lacking T cells display resistance to RGC loss following EIOP stress [12, 16, 18]. Although evidence regarding CD4^+^ T cells in human glaucoma is limited, elevated serum levels of pro-inflammatory cytokines associated with CD4^+^ T cells have been reported [17, 19–21].

Taken together, existing evidence strongly supports the involvement of CD4^+^ T cells in the pathogenesis of glaucoma. Given the immune privilege of the retina and its protection by the BRB, which restricts the passage of molecules from the general circulation to the neural retina, circulating CD4^+^ T cells demonstrate limited recognition of the retina and their access is denied under normal physiological conditions. Thus, unraveling the functional changes of circulating CD4^+^ T cells and the BRB, as well as the mechanisms enabling circulating CD4^+^ T cell invasion into the retina, represents the key to understanding the etiology of glaucoma.

In our current study, we utilized clinical samples, an EIOP-induced glaucoma mouse model with antibody-mediated treatments, and T cell transfer models, revealing the following findings: (i) circulating CD4^+^ T cells from glaucoma patients exhibited significantly upregulated activation and developed Th1-biased responses. Importantly, these changes correlated with glaucomatous visual impairment in patients; (ii) CD4^+^ T helper 1 (Th1) cells infiltrated the retina, specifically targeting the RGC, and integrated into the resident pro-inflammatory glial network, contributing to progressive RGC loss in glaucomatous mice; (iii) vascular cell adhesion protein 1 (VCAM-1), an endothelial adhesion molecule, was upregulated on retinal microvessels by circulating CD4^+^ Th1 cells, facilitating their influx into the neural retina; (iv) circulating CD4^+^ Th1 cells underwent functional reprogramming, acquiring a phenotype associated with lymphocyte migration and neurodegenerative diseases before reaching the retina. Through the lens of both peripheral immunity and the BRB, our findings unveil a novel perspective on how peripheral CD4^+^ T cells contribute to the pathogenesis of glaucoma and provide an opportunity to develop innovative strategies for intervening in this blinding disease.

## Materials and methods

### Participants

This study enrolled two cohorts of patients diagnosed with glaucoma (referred to as GL cohort #1, *n* = 96; GL cohort #2, *n* = 126) from April 2019 to March 2023. Circulating CD4^+^ T cell activation status was examined in GL-cohort #1 and functional subsets of CD4^+^ T cells were examined in GL-cohort #2. As previously reported [13, 14], the diagnosis of glaucoma was established by skilled ophthalmologists based on thorough ophthalmic examinations, considering factors such as age, family history, and clinical manifestations indicative of glaucomatous pathology. A comprehensive set of ophthalmic assessments was conducted, including evaluations of the anterior chamber angle, IOP measured on multiple occasions (with resulting averages recorded), cup-to-disk ratio, visual field loss, and retinal nerve fiber layer (RNFL) thickness. In accordance with established inclusion criteria, enrolled participants were without secondary glaucoma or any other visual disorders, had no undergone intraocular surgery within the past six months, exhibited no hematopoietic system diseases, systemic diseases (such as hypertension, diabetes, infections, systemic autoimmune diseases, and cancers), or other neurodegenerative disorders (such as Parkinson's disease and Alzheimer's disease). The extent of glaucomatous damage in patients was classified using the Hodapp, Parish, and Anderson (H-P-A) classification system. Two cohorts of age- and gender-matched healthy controls (HC-cohort #1, *n* = 98, for comparison with GL-cohort #1; HC-cohort #2, *n* = 121, for comparison with GL-cohort #2) who participated in annual health screenings at Sichuan Provincial People's Hospital were consecutively enrolled during the study. These individuals had no clinical evidence of glaucoma and no family history of the disease. Exclusion criteria: subjects reporting eye discomfort, elevated IOP (≥ 21 mmHg), or a history of other ocular diseases (such as uveitis, age-related macular degeneration, cataracts, high myopia, and retinitis pigmentosa). Additionally, individuals who had recently undergone surgery, presented with conditions affecting the immune system (such as hypertension, diabetes, infections, systemic autoimmune diseases, and cancers), exhibited other neurodegenerative disorders (such as Parkinson's disease and Alzheimer's disease), or were taking systemic medications impacting the immune system were also excluded.

### Human PBMC preparation

Peripheral blood mononuclear cells (PBMC) were collected from glaucoma patients and HC, following previously established protocols [13, 22]. In brief, fresh peripheral venous blood (10 ml) was obtained from each participant and promptly transported to the laboratory for PBMC isolation. The blood samples were diluted with Ca2^+^/Mg2^+^-free phosphate-buffered saline (PBS) and overlaid onto Ficoll-Paque™ PLUS (GE Healthcare Bioscience) in a 50 ml centrifuge tube. PBMC were purified by density gradient centrifugation, utilizing a centrifuge set at 2000 rpm for 20 min at 20 °C. The purified PBMCs were subsequently washed with PBS and Stain Buffer containing BSA (BD Bioscience). Flow cytometric staining was immediately performed to characterize the phenotypes of PBMCs.

### Mice and cell preparation

Wild type (WT) male C57BL/6 mice (10–12 weeks old) were purchased from the Beijing Vital River Laboratory Animal Technology Co., Ltd (Beijing, China). Mice with CD4-specific expression of Tomota (mice^CD4−V5−Tdtomota^ on a C57BL/6 background) purchased from the Shanghai Model Organisms (Shanghai, China). All mice were raised and bred in our facility under specific pathogen-free (SPF) conditions and under a 12/12 h dark/light cycle. Food and water were available *ad lib*. Single-cell preparation from retinas and peripheral blood was conducted following established protocols [23]. For retinal single-cell preparation, fresh retinas were isolated and incubated in RPMI 1640 medium supplemented with FBS and collagenase (5 mg/ml) at 37℃ for 20 min. Subsequently, gradient centrifugation using a 70/30% Percoll solution was carried out. Mouse peripheral blood was collected via cardiac puncture, and PBMC were purified. Finally, the isolated cells were resuspended in Stain Buffer containing bovine serum albumin (BSA) from BD Biosciences for subsequent analysis.

### EIOP-glaucoma model

To establish the EIOP-glaucoma model, male mice aged 8–12 weeks were utilized, following a previously reported protocol [24, 25]. Anesthesia was induced using 4% chloral hydrate. Pupil dilation was achieved by administering Compound Tropicamide Eye Drops (0.5%), while local anesthesia was provided using Oxybuprocaine Hydrochloride Eye Drops (0.4%). To induce EIOP, we injected 1.0 × 10^4^ polystyrene microbeads (1 μL) and triblock copolymer hydrogels (1 μL/eye) into the anterior chamber of both eyes. To prevent reflux, we promptly applied eye ointment and maintained the glass micropipette in place for 2 min before gently removing the needle. The microbeads employed in our study possessed a uniform diameter of 15 μm (Invitrogen). As controls, PBS (2 μL) was injected into the anterior chamber of both eyes of age- and gender-matched littermates. Following the procedure, mice were placed in a small animal incubator to allow for recovery. Subsequently, awake mice were gently positioned in a tube, secured in a plastic cone holder, and placed on a platform for intraocular pressure (IOP) measurements. Mice were given time to acclimate to the holder's position, and IOP readings were obtained at designated time points using a TonoLab tonometer (Colonial Medical Supply). Each set of measurements consisted of three consecutive readings, with six measurements per eye, which were then averaged to determine the IOP of the respective eye. For evaluation of glaucomatous mice, MB-injected mice with IOP values of both eyes ≥ 25 mmHg after modeling were used for further analysis (for those sacrificed on day 10, at least 1 measurement of IOP ≥ 25 mmHg within 10 days; for those sacrificed on/after day 20, at least 3 measurements of IOP ≥ 25 mmHg within 20 days) [25].

### In vivo antibody treatment

I) For CD4^+^ T cell depletion, intraperitoneal injection of Ultra-LEAF™ Purified anti-mouse CD4 Antibody (10 μg/g, clone GK1.5, BioLegend, San Diego, CA, USA) or corresponding isotype antibody (Ultra-LEAF Purified Rat IgG2b, κ Isotype Ctrl Antibody, clone RTK4530, 10 μg/g, BioLegend) was performed in mice on day 15, 25, and 40 PMI. ii) For CXCR3 blockade, intraperitoneal injection of Ultra-LEAF™ Purified anti-mouse CD183 (CXCR3) Antibody (10 μg/g, clone CXCR3-173, BioLegend) or corresponding isotype antibody (Armenian Hamster IgG, clone: HTK888, BioLegend) was performed in mice every 7 days since day 15 PMI. iii) For systemic IFN-g blocking, mice were injected i.p. with 200 mg of anti-IFN-g antibody (InVivoMAb anti-mouse IFNγ, BioXcell, clone XMG1.2) every 3 days since day 15 PMI. An IgG1 isotype antibody (HRPN, BioXcell) was used to serve as control. iv) To block VCAM-1, a InVivoMab anti-mouse VCAM-1 (10 mg/kg, BioXcell; corresponding isotype antibody: InVivoMAb rat IgG1 isotype control, clone HRPN, BioXcell) was administrated intravenously in MB-injected mice every 3 days since day 15 PMI. For retinal evaluation in abovementioned in vivo antibody intervention experiments, one retina from each recipient mouse was randomly selected and used for RGC density, while the other was for Iba1 and GFAP staining.

### Flow cytometry

Flow cytometric analysis was performed on freshly isolated single cells from human or mouse samples, following established protocols [13, 23]. For each flow cytometric test, 10^6^ cells were enumerated and collected in a FACS tube with a volume of 5 ml. These cells were then resuspended in 2 ml of Staining Buffer and centrifuged at 350*g*, 4 °C for 8 min. After removing the supernatant, the cells were incubated in the dark with 20 μL of Staining Buffer containing a viability dye (LIVE/DEAD™ Fixable Near-IR Dead Cell Stain Kit, Invitrogen, Thermo Fisher Scientific, USA) and fluorochrome-conjugated antibodies specific for the markers of interest for 30 min at 4°C. Following the incubation period, the cells were washed with 2 ml of Stain Buffer (BD Bioscience) and centrifuged at 350*g*, 4 °C for 8 min to collect the stained cells. To fix the stained cells, 200 μL of 1% paraformaldehyde was added for resuspension. Flow cytometric data were acquired immediately or stored in the dark at 4°C for up to 3 days and analyzed using a FACS Canto II flow cytometer (BD Biosciences) and FlowJo software (Tree Star, Ashland, OR, USA), respectively. The fluorochrome-conjugated antibodies used in this study included CD4 (mouse: RM4-5; human: RPA-T4; BD Bioscience, San Diego, CA, USA), CD3 (mouse: REA641; human: BW264/56; Miltenyi Biotec, Bergisch Gladbach, Germany), CXCR3 (mouse: S18001A; human: G025H7, BioLegend), CCR4 (human: L291H4, BioLegend), CCR6 (human: G034E3, BioLegend), CCR7 (human: G043H7, BioLegend),, CD45RO (human: UCHL1, BioLegend), CD11b (mouse: M1/70.15.11.5, Miltenyi Biotec), and CD45 (mouse: I3/2.3, BD Bioscience), CD62L (mouse: MEL-14, BioLegend), IFN-γ (mouse: XMG1.2, BioLegend), β1 (mouse: HMβ1-1, BioLegend). Isotype controls were included to exclude nonspecific binding.

### Whole mount retina preparation and immunohistochemistry

Whole-mount retinas were prepared following established procedures [23]. In brief, mouse eyes were carefully extracted, and the retinas were promptly dissected. Only one retina per mouse was used for each marker, and the other retina was utilized for staining of other marker or for RNA extraction or flow cytometry analysis. Subsequently, the retinas were immersed in a blocking solution comprising PBS with 0.1% Triton X-100 (v/v) and supplemented with 10% donkey serum (v/v). Primary antibodies were then applied to the whole-mount retinas and incubated overnight at 4 ℃. Following incubation, the retinas were thoroughly washed with PBS at least three times before being exposed to the corresponding secondary antibodies overnight at 4 ℃. Nuclei were counterstained using DAPI. The primary and secondary antibodies utilized in this study can be found in Additional file 1: Table S2. Confocal images were acquired using the LSM 800 confocal laser microscope (Zeiss, Germany). Throughout the imaging process, investigators were blinded to the experimental groups to ensure unbiased assessment. Quantitative analysis was conducted utilizing ImageJ software (version 3.1, National Institutes of Health, Bethesda, Washington).

### Adoptive transfer

Th1 cells were isolated through fluorescence-activated cell sorting (FACS) to ensure their purity. In brief, total splenic cells, as described earlier, were collected and subsequently stained with specific antibodies, including CD4 (RM4-5BD Bioscience), CD3 (REA641, Mitenyi Biotec), and/or CXCR3 (S18001A, BioLegend). The FACSAriaIII cell sorter (BD Biosciences) was employed for precise cell sorting. To facilitate tracking and identification, the sorted cells were labeled using the CellTrace CFSE Cell Proliferation Kit before their adoptive transfer. Subsequently, CFSE-labeled Th1 cells (2 × 10^6^ cells per recipient mice) were delivered into the designated recipient mice via tail vein injection, following established protocols [26].

### RNA isolation, quantitative real-time PCR, and RNA-seq

Total RNA was extracted from the retinas using TRIzol reagent (Invitrogen, CarIsbad, CA, USA) following the manufacturer's instructions. Subsequently, the RNA was reverse-transcribed using an RT reagent kit (Takara, Japan) to generate complementary DNA (cDNA). This cDNA served as the template for amplifying the target genes. To quantify gene expression, quantitative real-time PCR (qRT-PCR) was performed. A SYBR PrimeScript RT kit (Takara) and a 7500 Real-Time PCR system (Applied Biosystems) were employed for this purpose. The amplification protocol consisted of an initial denaturation step at 94 °C for 5 min, followed by 40 cycles of denaturation at 94 °C for 30 s, annealing at 60 °C for 30 s, extension at 72 °C for 30 s, and a final extension step at 72 °C for 10 min. The 2^−DCt^ method was utilized for quantification of transcript levels, with β-actin serving as the reference gene for normalization.

For RNA-seq, total RNA was extracted from target cells as described above. The standard Illumina RNA-seq protocol was followed for library preparation. The prepared libraries underwent sequencing on an Illumina Novaseq 6000 platform, generating 150 bp paired-end reads. Subsequently, in-house Perl scripts were initially employed to process the raw data (raw reads) in fastq format and obtain high-quality clean data. The reference genome index was constructed using Hisat2 v2.0.5, and the paired-end clean reads were aligned to the reference genome using Hisat2 v2.0.5. Differential expression analysis between the two groups was conducted using the edgeR R package (version 3.22.5). The P-values were adjusted using the Benjamini & Hochberg method. A threshold for significant differential expression was set at a corrected P-value of 0.05 and an absolute fold change of 2. Gene ontology (GO) enrichment analysis of the differentially expressed genes was performed using the cluster Profiler R package, which corrects for gene length bias. GO terms with a corrected P-value below 0.05 were considered significantly enriched by the differentially expressed genes.

### Statistical analysis

All statistical analyses in this study were performed using the Prism 8.4 software (GraphPad Software Inc., San Diego, CA). To assess data normality, the Kolmogorov–Smirnov test was employed. For non-normally distributed data, the Mann–Whitney test was utilized to compare differences between two groups. In cases where there were three or more groups, the Kruskal–Wallis test was conducted to evaluate differences among the groups. Subsequently, the Dunn's multiple comparisons test was employed to determine the specific differences between each group and every other group. For normally distributed data, a two-tailed unpaired Student's t-test was utilized to compare two groups, while one-way ANOVA followed by Tukey's multiple comparisons test was applied when there were three or more groups. The significance level for all statistical analyses was set at 5% (*P* < 0.05).

## Results

### Circulating CD4^+^ T cell response is enhanced with the visual damage stage in patients with glaucoma

**Fig. 1:**
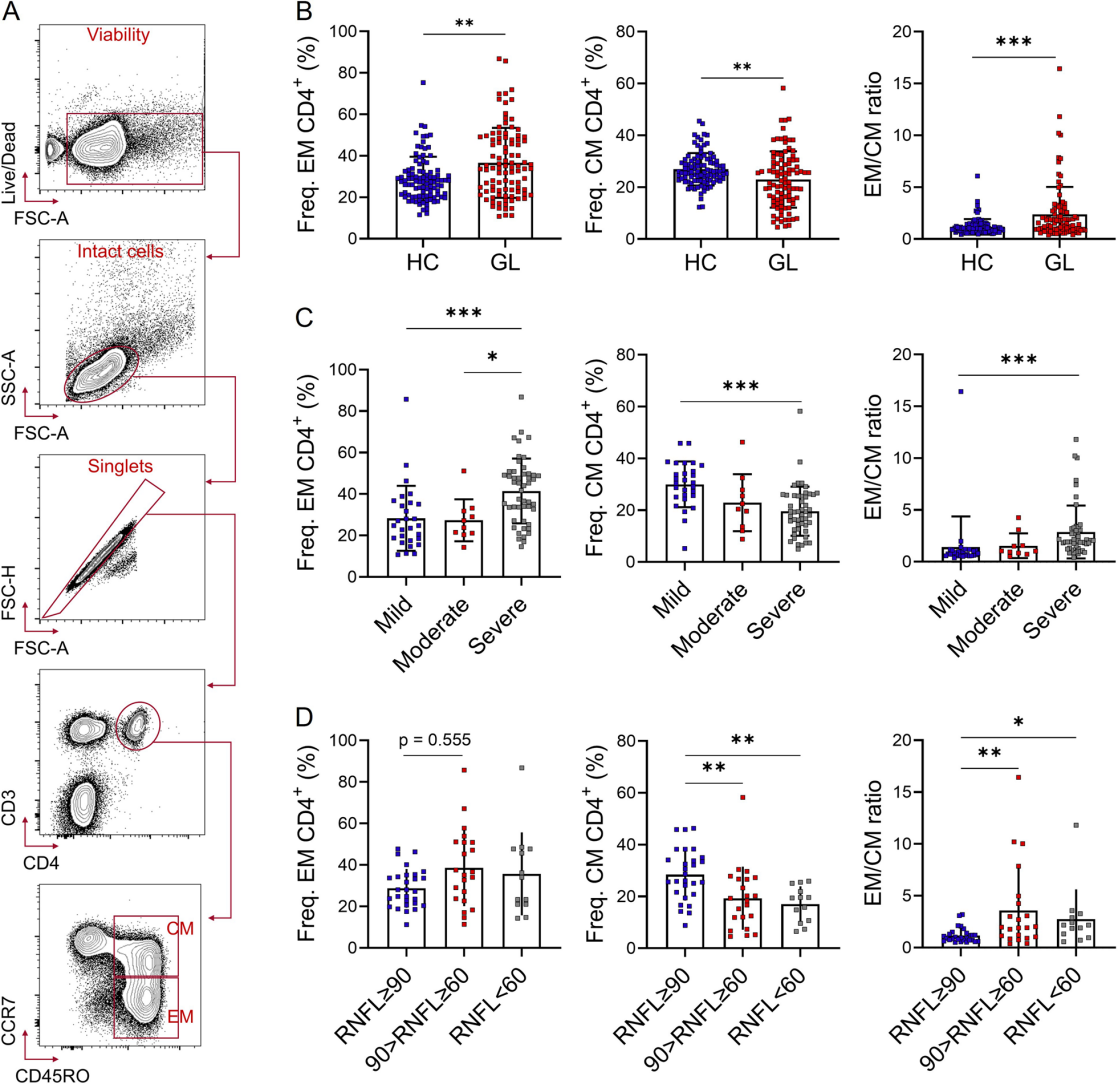
Circulating CD4^+^ T cell response is enhanced with the visual damage stage in patients with glaucoma. **A** Flow cytometric gating strategy for peripheral blood mononuclear cells (PBMC). PBMC were isolated from glaucoma patients (GL) and healthy controls (HC) enrolled in this study. A LIVE/DEAD™ Fixable Near-IR Dead Cell Stain Kit was used to determine the viability of PBMC. CD3 and CD4 were used to determine CD4^+^ T cells. CCR7 and CD45RO were used to determine the activation status of CD4^+^ T cells. Representative flow cytometric plots were shown. **B** Comparison of the frequency (Freq.) of peripheral blood effector memory (EM), central memory (CM) CD4^+^ T cells, and the EM-to-CM ratio (EM/CM) between GL (*n* = 87) and HC (HC, *n* = 98). **C**, **D** Shown are comparison of Freq. EM CD4^+^, CM CD4^+^, and EM/CM among different groups stratified according to **C** visual damage stage based on H-P-A classification system (Mild, *n* = 28; Moderate, *n* = 10; Severe, *n* = 49) and **D** retinal nerve fiber layer (RNFL) thickness (RNFL ≥ 90 mm, *n* = 28; 60 ≤ RNFL < 90 mm, *n* = 22; RNFL < 60 mm, *n* = 13), respectively. *P < 0.05, **P < 0.01, ***P < 0.001. Statistical comparisons were performed using **B**, Mann–Whitney test; **C**, **D**, Kruskal–Wallis test followed by Dunn's multiple comparisons test

While a few studies have sporadically observed alterations in peripheral blood T cells in patients with glaucoma [19–21], the activation status of CD4^+^ T cells in glaucoma remains poorly understood. According to the global standardization of flow cytometric immunophenotyping for human [27], CD4^+^ T cells can be categorized into different subsets, mainly including naïve, central memory (CM CD4^+^), and effector memory (EM CD4^+^), based on their activation status (Fig. 1A). Here, we recruited a cohort of glaucoma patients and healthy donors, providing detailed demographics and clinical parameters in Table S1. Remarkably, we discovered a significant increase in the frequency of peripheral blood EM CD4^+^ (Freq. EM CD4^+^) among total CD4^+^ T cells in glaucoma patients, alongside a significant decrease in Freq. CM CD4^+^ compared to healthy donors (Fig. 1B). Moreover, the ratio of EM CD4^+^-to-CM CD4^+^ (EM/CM) was notably higher in glaucoma patients, indicating a transformation of CD4^+^ T cells from a " quiescent" to a " primed for action" state. Additionally, patients were stratified into three groups based on the Hodapp, Parish, and Anderson (H-P-A) classification system: Early, Moderate, and Severe. Notably, individuals with higher Freq. EM CD4^+^, EM/CM, or lower Freq. CM CD4^+^ exhibited more severe visual damage (Fig. 1C). Furthermore, we observed a positive correlation of EM/CM with both retinal nerve fiber layer (RNFL) thinning (Fig. 1D) and cup-to-disc ratio (C/D) enlargement, practical parameters indicative of glaucomatous neural damage (Additional file 1: Fig. S1). These data suggest a positive association between CD4^+^ T cell activation and the severity of glaucoma.

### Th1 response is predominant in patients with glaucoma

**Fig. 2:**
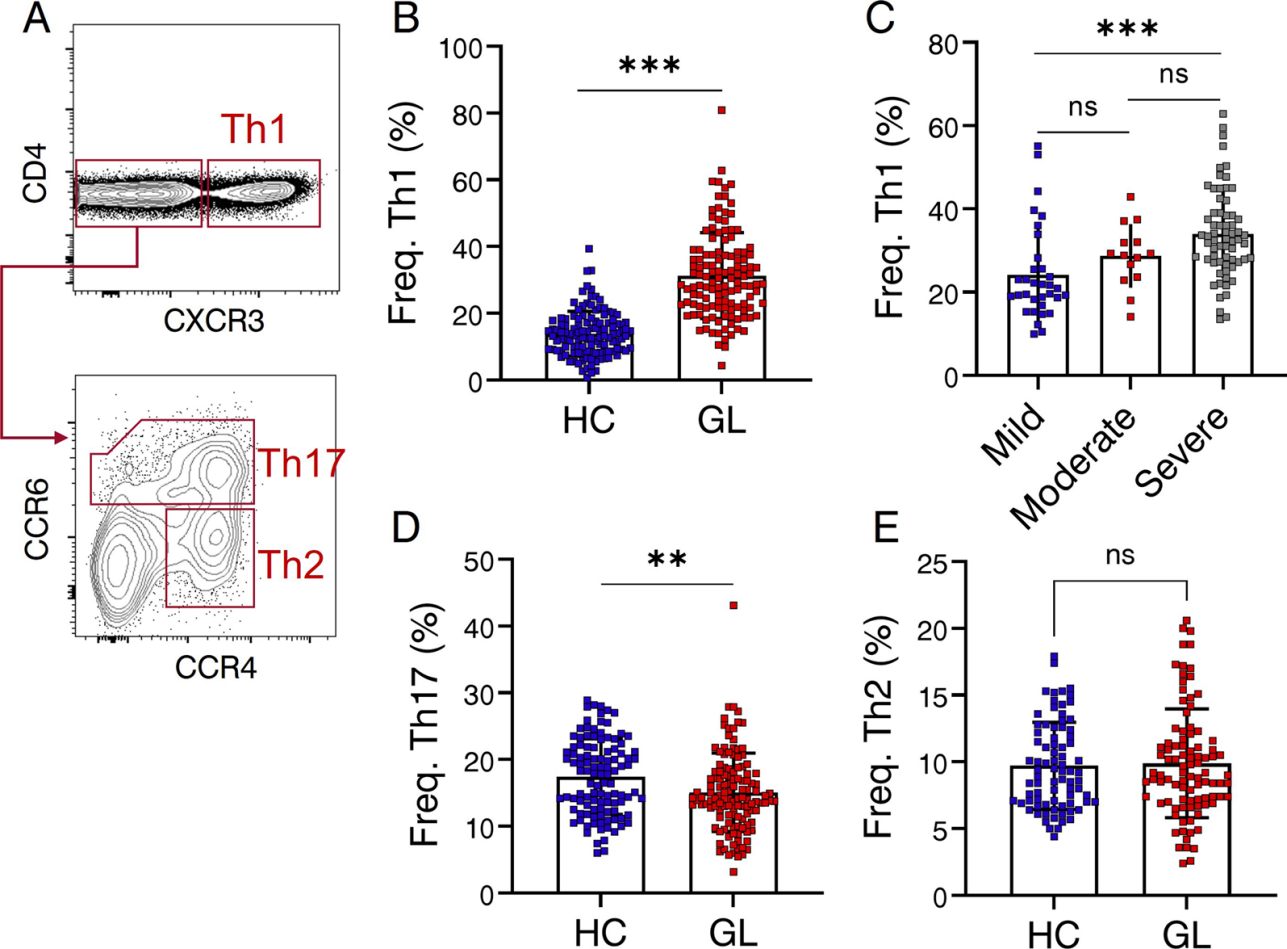
T helper 1 (Th1) response is predominant in patients with glaucoma. **A** Flow cytometric gating strategy to identify CD4^+^ T cell subsets in PBMC. PBMC were collected from GL and HC. CXCR3, CCR6 and CCR4 were used to determine the Th1 (CXCR3^+^), Th2 (CXCR3^−^ CCR6^−^ CCR4^+^), and Th17 (CXCR3^−^ CCR6^−^ CCR4^+^) subset of live CD4^+^ T cells. Representative flow cytometric plots were shown. **B** Comparison of Freq. Th1 between GL (*n* = 113) and HC (HC, *n* = 121). **C** Shown is comparison of Freq. Th1 among different groups stratified according to visual damage stage based on H-P-A classification system (Mild, *n* = 33; Moderate, *n* = 14; Severe, *n* = 66). **D**, **E** Comparison of **D** Freq. Th17 and **E** Freq. Th2 between GL and HC. **P < 0.01, ***P < 0.001, ns, no significance. Statistical comparisons were performed using **B**, **D**, **E** Mann–Whitney test; **C**, Kruskal–Wallis test followed by Dunn's multiple comparisons test

Chemokine receptors have proven useful in delineating distinct migratory capacities and effector functions of human T cell subsets. Not only do these receptors form the basis for differentiating activation states between CM and EM CD4^+^ subsets, but they also allow for the identification and isolation of T-cell subsets with varying effector functions, and tissue tropisms. CD4^+^ T cells can be further defined as Th1 (CXCR3^+^), Th2 (CXCR3^−^ CCR6^−^ CCR4^+^), and Th17 (CXCR3^−^ CCR6^−^ CCR4^+^) subsets (Fig. 2A) [27]. Remarkably, the Th1 subset was significantly increased in the peripheral blood of glaucoma patients compared to control subjects (Fig. 2B). Additionally, patients with severe glaucomatous visual damage demonstrated higher frequencies of Th1 cells compared to those in the mild group (Fig. 2C). Conversely, the Th17 subset displayed an inverse distribution pattern when compared to their Th1 counterparts (Fig. 2D), while no noticeable difference in the frequency of Th2 cells was observed between patients and controls. Collectively, these findings establish a correlation between Th1 cells and the development of glaucoma.

### Peripheral Th1 cells are required for glaucomatous RGC loss

**Fig. 3:**
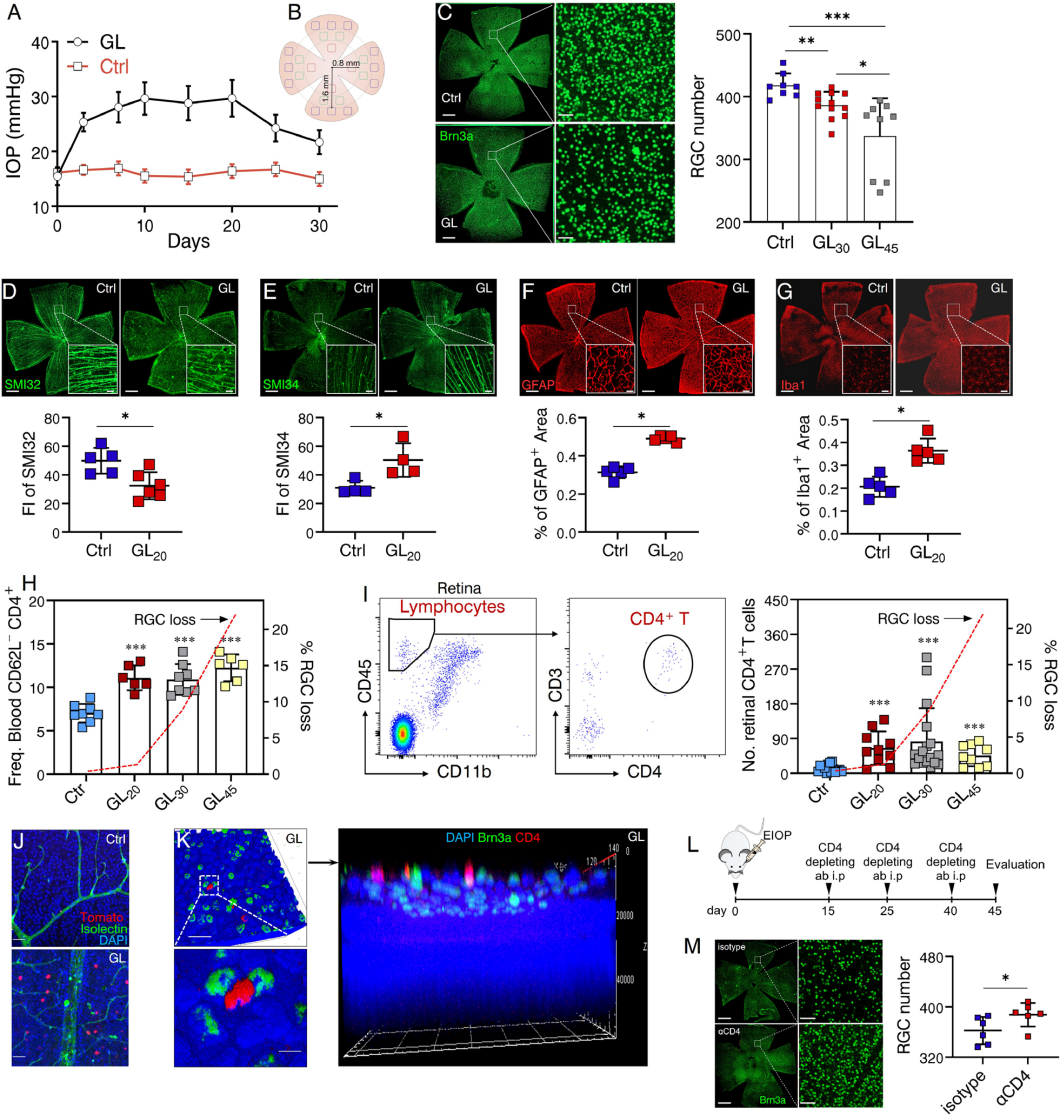
CD4^+^ T cells are required for glaucomatous RGC loss. An elevated intraocular pressure (EIOP)-induced glaucoma model was established after a single microbead (MB) injection. PBS-injected mice served as control (Ctr). **A** Pooled data from three independent experiments conducted to determine the IOP curve (the total number of enrolled mice: MB, *n* = 30; Ctr, *n* = 30). **B**, **C** MB-injected mice were sacrificed and retinas were collected at day 30 (GL_30_) and 45 (GL_45_) after MB injection, respectively. Immunofluorescence staining for Brn3a (used as a RGC marker) was performed to quantify RGC number, presented as the loss in relative to Ctr retinas. **B** Illustration of the six areas (each area size: 0.1 mm^2^) that were examined as indicated in each quadrant of one retina flat-mount. Red boxes represent the central areas, green boxes represent the middle-peripheral areas, and blue boxes represent the peripheral areas. Brn3a-labeled RGCs per 0.1mm^2^ area in one retina flat-mount from each mouse was quantified in 4 quadrants, and the average loss of six areas was also calculated. **C**–**H** Shown are results representing one of three independent experiments. **C** Representative Brn3a-stained retina flat-mounts and RGC number is shown (scale bar: left, 500 μm; right, 20 μm). Sample size: Ctr, *n* = 8; GL_30_, *n* = 12; GL_45_, *n* = 9. *n* refers to the number of retinas used for RGC density analysis and one retina was analyzed per mouse. *P < 0.05, ***P < 0.001, Kruskal–Walls test followed by Dunn’s multiple comparisons test. **D**–**G**, Retina tissues were collected from MB-injected mice on day 20 (GL_20_) or Ctr (scale bar: 500 μm; inset, 20 μm). Retina flat-mounts were prepared and stained for **D** SMI32, **E** SMI34, **F** GFAP, and **G** Iba1. For determination of **D** SMI32 and **E** SMI34 expression, fluorescence intensity (FI) was quantified. For determination of **F** GFAP and **G** Iba1, the percentage of antibody-labeled areas per microscopic field (size: 319.45 μm^2^) was quantified. **D-G** *n* ≥ 4. *n* refers to the number of retinas used for SMI32, SMI34, GFAP, or Iba1 staining. Only one retina per mouse was used for each marker. **H** PBMC were collected from mice (Ctr, *n* = 8; GL_20_, *n* = 6; GL_30_, *n* = 8; GL_45_, *n* = 6. *n* refers to the number of enrolled mice) and flow cytometry was performed to determine the difference of Freq. CD62L^−^ CD4^+^ T cells in the blood among indicated groups. The kinetics of RGC loss (the red-dashed line, derived from **C**, right Y axis) is also shown. **I** Pooled data from multiple independent experiments are shown (the total number of mice in each group: Ctr = 60; GL_20_ = 40; GL_30_ = 68; GL_45_ = 36). Retinal cells were collected from Ctr or GL mice at indicated timepoints. Flow cytometry was performed to determine the number of retinal CD4^+^ T cells. (left) Representative flow cytometric plots were shown. (right) Quantification of retinal CD4^+^ T cells (cell number in every 10^5^ live retinal cells) in Ctr (*n* = 15), GL_20_ (*n* = 10), GL_30_ (*n* = 17), and GL_45_ (*n* = 9). *n* refers to the number of flow cytometry tests. Four retinas from 4 individual mice (one retina from each mouse) were combined for each flow cytometry test. **J** Shown is representative immunofluorescence staining for DAPI and Isolectin in retina flat-mounts from GL mice with CD4-specific expression of Tomato (a red fluorescent protein, scale bar: 20 μm) (3 independent experiments were conducted, 4 mice per group in each experiment). **K** Representative immunofluorescence staining (from 8 mice) for DAPI, CD4, and Brn3a in retina flat-mounts from GL mice showing the location of CD4^+^ T cells with the Brn3a^+^ RGC layer (scale bar: upper and right, 20 μm; lower, 5 μm). **L** Experimental design: glaucoma was induced and mice were intraperitoneally injected with anti-CD4 (aCD4) or isotype antibody on day 15, 25, 40 after MB injection. **M** Shown are results representing one of three independent experiments and RGC number was evaluated (*n* = 6 each group. *n* refers to the number of retinas used for RGC density analysis and one retina was analyzed per mouse) as described in **C**. **H**, **I** ***P < 0.001 versus Ctr group, Kruskal–Walls test followed by Dunn’s multiple comparisons test. **D**–**G**, **M** **P < 0.01, two-tailed unpaired Student’s* t* test was performed

In order to gain deeper insights into the involvement of CD4^+^ T cells in glaucomatous retinal neurodegeneration, we established an EIOP-induced glaucoma model characterized by an approximately 28-day EIOP period followed by prolonged RGC loss [25, 28]. EIOP was induced by injecting microbeads (MB) suspension into the anterior chamber of both eyes (Fig. 3A), while phosphate-buffered saline (PBS) injection served as the control. RGC loss associated with glaucoma was assessed by immunofluorescence staining of retinal flat-mounts for Brn3a, a specific marker for RGCs (Fig. 3B, C). Statistical analysis revealed significant RGC loss after 30 days of modeling. Notably, neuroinflammation and early RGC injury were observed even before day 30 following MB injection. Axon injury in RGCs was assessed by decreased SMI32 expression (a non-phosphorylated neurofilament protein, Fig. 3D) and increased SMI34 expression (a phosphorylated neurofilament protein, Fig. 3E). Increased presence of GFAP^+^ (astrocytes, Fig. 3F) and Iba1^+^ cells (microglia, Fig. 3G) was observed in glaucomatous retinas compared to control retinas, indicating heightened gliosis and neuroinflammation. Furthermore, we aimed to investigate the relationship between circulating CD4^+^ T cells and RGC loss in glaucomatous mice. Consistent with observations in human patients, activation of circulating CD4^+^ T cells was significantly upregulated in glaucomatous mice (Fig. 3H). Notably, CD4^+^ T cells had displayed an activated phenotype on day 20, preceding the onset of significant RGC loss (Fig. 3H). Moreover, flow cytometry analysis demonstrated the infiltration of CD4^+^ T cells into the retina on day 20 (Fig. 3I), suggesting their potential involvement in initiating progressive RGC loss. The presence of CD4^+^ T cells in glaucomatous retinas was further confirmed using mice with CD4-specific expression of Tomato, a red fluorescent protein (Fig. 3J). Notably, the localization of CD4^+^ T cells within the RGC layer was also evidenced (Fig. 3K). To gain further insight into the role of CD4^+^ T cells, we performed antibody-mediated depletion of systemic CD4^+^ T cells in glaucomatous mice (Fig. 3L). As expected, anti-CD4 treatment significantly attenuated RGC loss (Fig. 3M) and GFAP expression (Additional file 1: Fig. S2) in glaucomatous retinas compared to isotype controls.

**Fig. 4:**
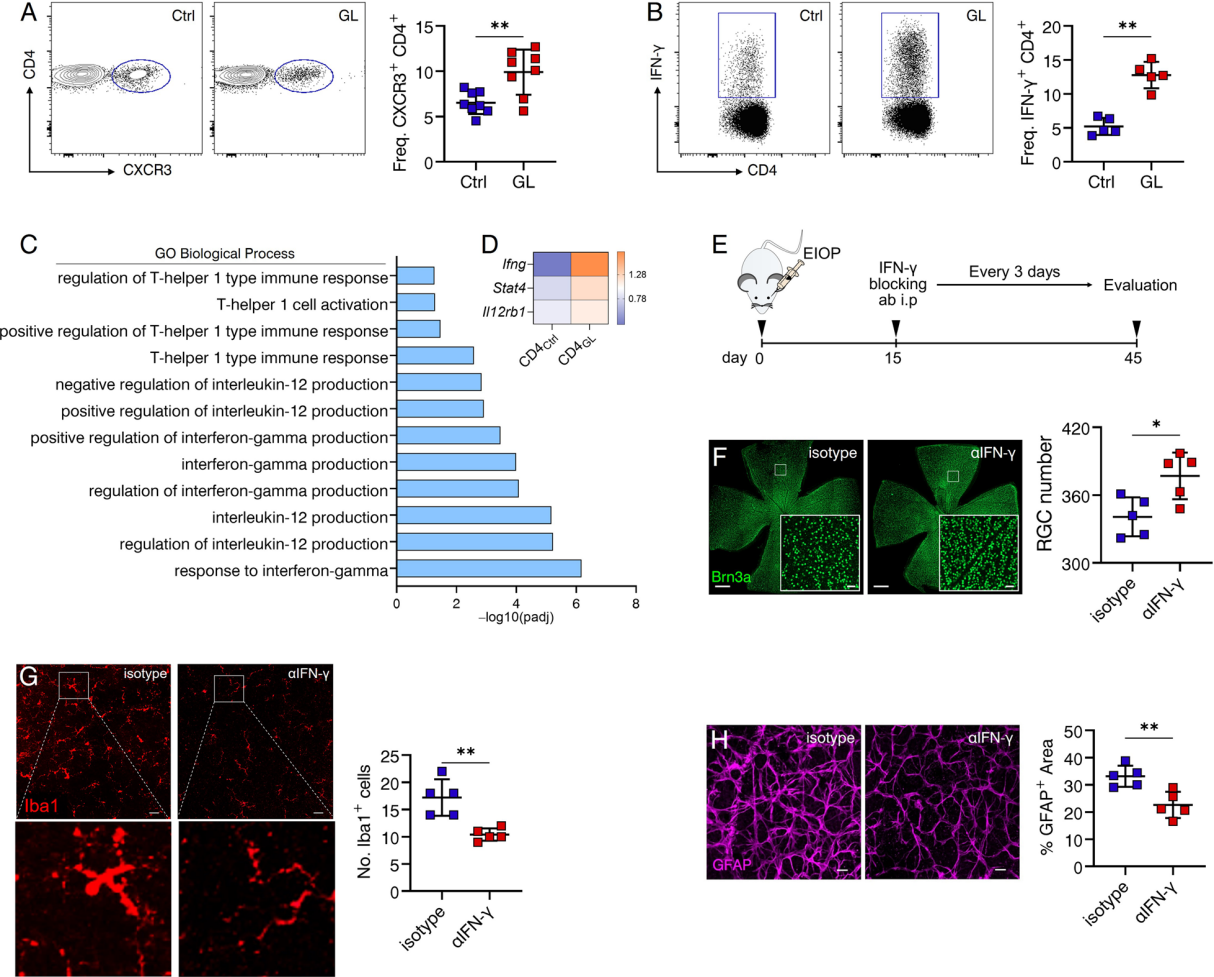
Block of Th1 response ameliorates glaucomatous retinal degeneration. Flow cytometry was performed to determine circulating **A** Freq. CXCR3^+^ CD4^+^ T cells (Ctr, *n* = 8; GL, day 20, *n* = 8) and **B** Freq. IFN-g^+^ CD4^+^ T cells (Ctr, *n* = 5; GL, day 20, *n* = 5). **A**, **B** Shown are results representing one of three independent experiments and *n* refers to the number of used mice. **C**, **D** Peripheral blood CD4^+^ T cells from GL_20d_ (CD4_GL_, *n* = 24) and Ctr mice (CD4_Ctr_, *n* = 24) were obtained by fluorescence-activated cell sorting (FACS). RNA-seq was performed to analyze the gene expression profile in CD4_GL_ and CD4_Ctr_. **C** Analysis of Gene Ontology (GO) enriched pathways showing significantly altered Th1-associated biological processes (BP) in CD4_GL_ compared to those in CD4_Ctr_. **D** Heatmap of differentially expressed genes (DEG) involved in Th1 differentiation. **E** Experimental design: glaucoma was induced and mice were intraperitoneally injected with anti-IFN-g (aIFN-g) or isotype antibody every 3 days from day 15 after MB injection to the end of indicated experiments. **F**, **H** Shown are results representing one of three independent experiments. In each experiment, one random retina from each recipient mouse was used for RGC density and the other was for Iba1 and GFAP staining. *n* = 5, *n* refers to the number of retinas for analysis. The number of **F** RGC, **G** microglia (magnification: morphological changes), and **H** the percentage of GFAP^+^ area per microscopic field (size: 319.45 μm^2^) was calculated. **F** scale bar: 500 μm; inset, 20 μm. **G-H** scale bar: 20 μm. **P < 0.01, two-tailed unpaired Student’s* t* test was performed

Having established the predominance of Th1 cells in patients with glaucoma (Fig. 2), their role in glaucomatous RGC loss was further investigated. First, the increase of circulating CXCR3-expressing (Fig. 4A) and IFN-γ-producing (Fig. 4B) CD4^+^ T cells in glaucomatous mice was validated. Furthermore, we collected CD4^+^ T cells from glaucomatous mice, referred to as CD4_GL_, and performed RNA-seq analysis to elucidate their gene expression profile. CD4^+^ T cells from control mice, referred to as CD4_Ctr_, were also analyzed for comparison. Our analysis, encompassing Gene Ontology (GO) enrichment and differentially expressed genes (DEG) assessment, revealed that CD4_GL_ exhibited altered biological processes (BP) with a greater enrichment of Th1-associated responses (Fig. 4C). Notably, the expression of Th1-associated genes was significantly upregulated in CD4_GL_ compared to CD4_Ctr_ (Fig. 4D). Collectively, these findings underscore the involvement of Th1 cells in glaucoma. Subsequently, we investigated whether circulating Th1 response contributes to glaucomatous retinal loss through systemic blockade of IFN-γ (Fig. 4E). Remarkably, this intervention led to a significant amelioration of RGC loss (Fig. 4F), microglial activity (Fig. 4G), and gliosis (Fig. 4H). In summary, these data provide support for the notion that Th1-mediated response may play a contributory role in glaucomatous retinal loss and neuroinflammation.

### Th1 cells aggregate microglia and deteriorate glaucomatous RGC loss

**Fig. 5:**
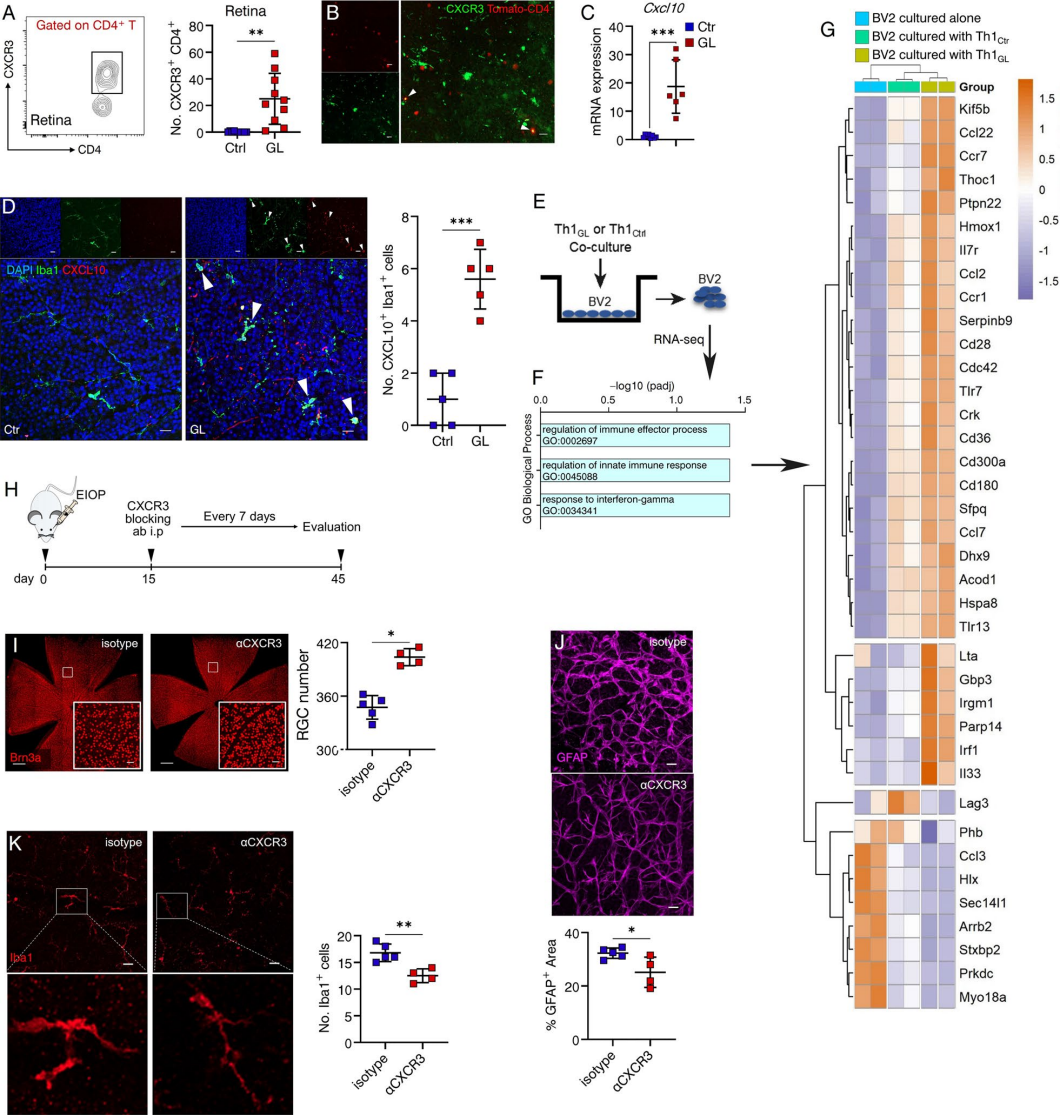
Th1 cells aggregate microglia and deteriorate glaucomatous RGC loss. **A** Pooled data from 3 independent experiments are shown. Flow cytometry was performed to quantify retina-infiltrating CXCR3^+^ CD4^+^ T cells of Ctr (*n* = 10) and GL (day 20, *n* = 10). *n* refers to the number of flow cytometry tests. Four retinas from 4 individual mice (one retina from each mouse) were combined for each flow cytometry test. **B** Representative confocal image of a whole-mount retina of a MB-injected mouse (from 8 mice) with CD4 specific expression of Tomota (red), stained for CXCR3 (green) (scale bar: 20 μm). **C**, **D** Shown are results representing one of three independent experiments. **C** qRT-PCR was performed to measure the retinal expression levels of *Cxcl10* mRNA. Ctr, *n* = 8; GL, *n* = 6. *n* refers to the number of RNA tests. Two retinas from 2 mice (one retina from each mouse) were combined for each RNA test. **D** Representative confocal image of a whole-mount retina of GL and Ctr mouse, stained for Iba1, CXCL10, and DAPI (scale bar: 20 μm). The number of Iba1^+^ CXCL10^+^ cells were quantified. *n* = *5, n* refers to the number of retinas used for Iba1 staining and one retina was analyzed per mouse. **E** Shown is a schematic of the cell co-culture design: Splenic CXCR3^+^ Th1 cells sorted by FACS from GL (cells pooled from 8 mice, day 20, referred to as Th1_GL_) and Ctr mice (cells pooled from 8 mice, referred to as Th1_Ctrl_) were co-cultured in vitro with BV2 cells (a mouse microglial cell line), respectively. After 48 h of co-culture, each well was washed to remove non-adhesive T cells. RNA-seq was performed to analyze the gene expression profile in remaining BV2 cells (two BV2 samples per group). **F** Immune response-associated pathway analysis of genes enriched in BV2 cells. **G** Heatmap of DEG involved in immune responses. **H** Experimental design: glaucoma was induced and mice were intraperitoneally injected with anti-CXCR3 (αCXCR3) or isotype antibody every 7 days from day 15 after MB injection to the end of indicated experiments. **I**–**K S**hown are results representing one of three independent experiments. In each experiment, one random retina from each recipient mouse was used for RGC density and the other was for Iba1 and GFAP staining (isotype, *n* = 5; αCXCR3, *n* = 4; *n* refers to the number of retinas for analysis). RGC number, **J** the percentage of GFAP^+^ area per microscopic field (size: 319.45 μm^2^), and **K** the number of microglia (magnification: morphological changes) were calculated. **I** scale bar: 500 μm; inset, 20 μm. **J**, **K** scale bar: 20 μm. *P < 0.05, **P < 0.01, ***P < 0.001, ns, no significance. two-tailed unpaired Student’s* t* test was performed

Next, the mechanistic role of Th1 cells in regulating RGC loss was elucidated in glaucomatous mice. Notably, employing surface chemokine receptors for the identification, quantification, and isolation of T cell lineages provides greater stability compared to the utilization of transcription factors or cytokine production. As depicted in Fig. 2, the surface chemokine receptor CXCR3 serves as a distinguishing marker for Th1 cells. It has been extensively-documented that CXCR3 is predominantly expressed by Th1 cells. Its expression is under the regulation of T-bet, the transcription factor associated with the Th1 cell lineage, and signaling through CXCR3 triggers Th1 cell-mediated inflammation [29]. Our investigation confirmed that nearly all IFN-γ-producing CD4^+^ T cells were CXCR3^+^ (Additional file 1: Fig. S3). Consequently, we conducted flow cytometric quantification, revealing a substantial increase in retinal Th1 cells in glaucomatous mice (Fig. 5A). Additionally, glaucoma was induced in mice with CD4-specific expression of Tomato, and immunofluorescence staining using anti-CXCR3 antibodies demonstrated a significant presence of CXCR3^+^ CD4^+^ T cells within the retina (Fig. 5B). Furthermore, retinal CXCL10, the ligand for CXCR3, exhibited a substantial upregulation (Fig. 5C), and CXCL10-producing microglia expanded concurrently with the presence of CXCR3^+^ Th1 cells in glaucomatous retinas (Fig. 5D), indicating a potential interaction between these two cellular units. Microglia are major resident innate immune cells within the retina and abnormal microglia activity has been implicated as an essential factor in the pathophysiology of glaucoma [4, 30]. In the central nervous system (CNS), it has been suggested that CD4^+^ T cells facilitate neurodegenerative disease pathology through interaction with microglia [31]. Therefore, we postulated that Th1 cells may engage in crosstalk with microglia, thereby promoting microglia pathology in glaucoma. We sorted CXCR3^+^ Th1 cells from glaucomatous mice (referred to as Th1_GL_) and control mice (referred to as Th1_Ctrl_) to perform in vitro co-culture with BV2 cells (a mouse microglial cell line) (Fig. 5E). After 48 h of co-culture, transcriptional analysis revealed that BV2 cells co-cultured with Th1_GL_ profoundly induced pathways associated with response to interferon-gamma, regulation of innate immune response and immune effector process (Fig. 5F), and upregulated several clusters of associated genes (Fig. 5G). These data suggest that Th1 cells directly augment microglia pathology in glaucoma, which could contribute to the development of progressive RGC loss. Furthermore, we proceeded to examine the functional significance of Th1 cells in retinal microglial activation in vivo. Mice subjected to EIOP were administered intraperitoneal anti-CXCR3 antibodies (Fig. 5H), resulting in a substantial amelioration of RGC loss (Fig. 5I) and marked suppression of gliosis (Fig. 5J) and microglial activity (Fig. 5K). Collectively, our findings suggest that Th1 cells deteriorate glaucomatous RGC loss, potentially through interactions with retinal microglia, thus fostering a pro-inflammatory milieu conducive to RGC loss.

### Circulating Th1 cells undergo phenotypic transformation before their infiltration into the retina in glaucoma

**Fig. 6:**
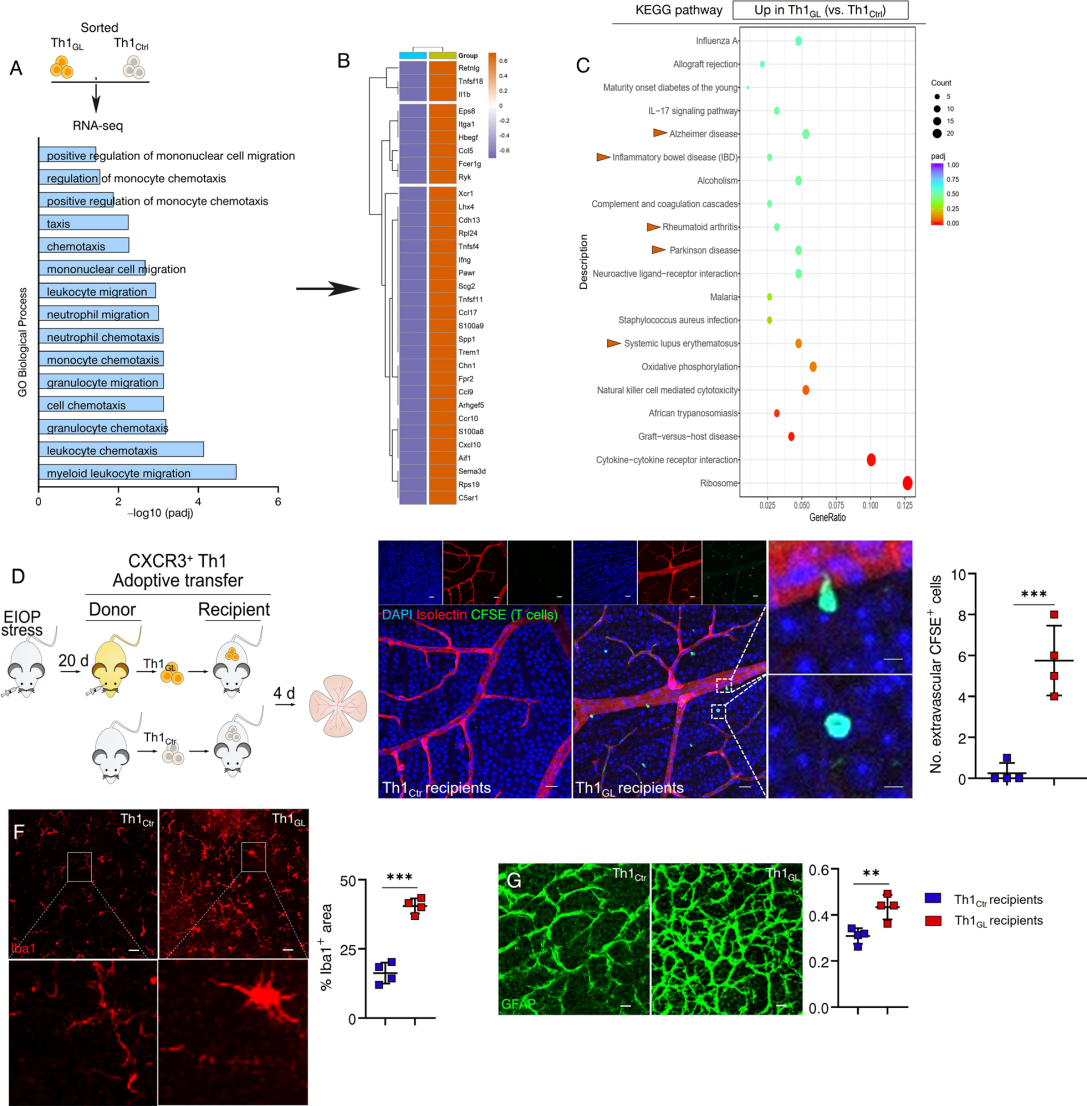
Circulating Th1 cells undergo phenotypic transformation before their infiltration into the retina in glaucoma. **A**–**C** Splenic Th1_GL_ (day 20 days after MB injection, cells pooled from 8 mice) and Th1_Ctr_ (cells pooled from 8 mice) were sorted by FACS and subjected them to transcriptomic analysis using RNA-seq. **A** The main category terms associated with cell migration in the biological process class of GO (Gene Ontology) analysis. **B** Heatmap of DEG involved in cell migration. **C** The main category terms of KEGG pathway analysis. **D** Experimental design: Th1_GL_ and Th1_Ctr_ were labeled with a CellTrace CFSE Cell Proliferation Kit, and then transferred into naïve WT recipient mice via tail vein (2 × 10^6^ cells per recipient mice), respectively. After 4 days of reconstitution, all recipients were sacrificed and their retinas were collected. **E**–**G** Shown are representative results of one of three independent experiments. In each experiment, one random retina from each recipient mouse was used to show Isolectin^+^ vessels and CFSE^+^ cells, and the other was for Iba1 and GFAP staining.* n* = 4. *n* refers to the number of retinas for analysis. **E** Representative confocal images of CFSE-labeled cells (green) in retina flat-mounts, stained for Isolectin (red) and DAPI (blue) (scale bar: 20 μm; inset, 5 μm). Quantification of extravascular CFSE-labeled cells (per microscopic field, size: 266.21 μm^2^) in retina flat-mounts is presented. In each flat-mount, at least 3 microscopic fields were analyzed in each quadrant and 4 quadrants were examined. **F**, **G** Retina flat-mounts of Th1_GL_ and Th1_Ctr_ recipients were also stained for **F** Iba1 and **G** GFAP. For determination of **F** Iba1 and **G** GFAP, the percentage of antibody-labeled areas per microscopic field (size: 319.45 μm^2^) was quantified. **F**, **G** scale bar: 20 μm. *P < 0.05, **P < 0.01, ***P < 0.001, two-tailed unpaired Student’s t test was performed

In the context of normal physiological conditions, circulating Th1 cells do not recognize the retina and exhibit no inclination to migrate towards it. Previous investigations have suggested the fact that only activated CD4^+^ T cells possess the capability to traverse the BRB from the circulation [32]. Hence, we embarked on an investigation to elucidate the mechanism underlying the phenotypic transformation of circulating Th1 cells, leading to their acquisition of a more "aggressive" phenotype, facilitating their infiltration into the retina in glaucoma. To this end, we purified Th1_GL_ and Th1_Ctr_, as described above, and subjected them to transcriptomic analysis using RNA-seq. Employing GO enrichment analysis, we observed significant alterations in BP within the Th1_GL_, showing a heightened enrichment in leukocyte chemotaxis and migration (Fig. 6A, B). Notably, The KEGG analysis revealed that Th1_GL_ cells exhibited pathway enrichment in neurodegenerative conditions such as Alzheimer's disease and Parkinson's disease, as well as inflammatory diseases including systemic lupus erythematosus, rheumatoid arthritis, and inflammatory bowel disease (Fig. 6C). This intriguing finding suggests that Th1 cells acquire a functional phenotype associated with migration, neuroinflammation, and neurodegeneration prior to their arrival at the retina, potentially catalyzing their pathogenicity in glaucoma. To validate this hypothesis, a T cell adoptive transfer model was established, in which Th1_GL_ or Th1_Ctr_ cells labeled with CFSE were transferred into EIOP-naïve recipient mice via the tail vein (Fig. 6D). After 4 days of reconstitution, we observed evident extravascular CFSE-labeled cells within the retinas of Th1_GL_ recipients (Fig. 6E). In contrast, Th1_Ctr_ recipients barely exhibited detection of CFSE-labeled cells within their retinas (Fig. 6E). As anticipated, upregulated microglial activation (Fig. 6F) and gliosis (Fig. 6G) were only evident in the retinas of Th1_GL_ recipients, but not in Th1_Ctr_ recipients. These results corroborate the notion that circulating Th1 cells in glaucoma exhibit a tropism for the retina and have the capacity to establish a communication network with the resident innate immune cells of the retina.

### Retinal endothelial expression of VCAM-1 is critical for Th1 cell migration across the BRB

**Fig. 7:**
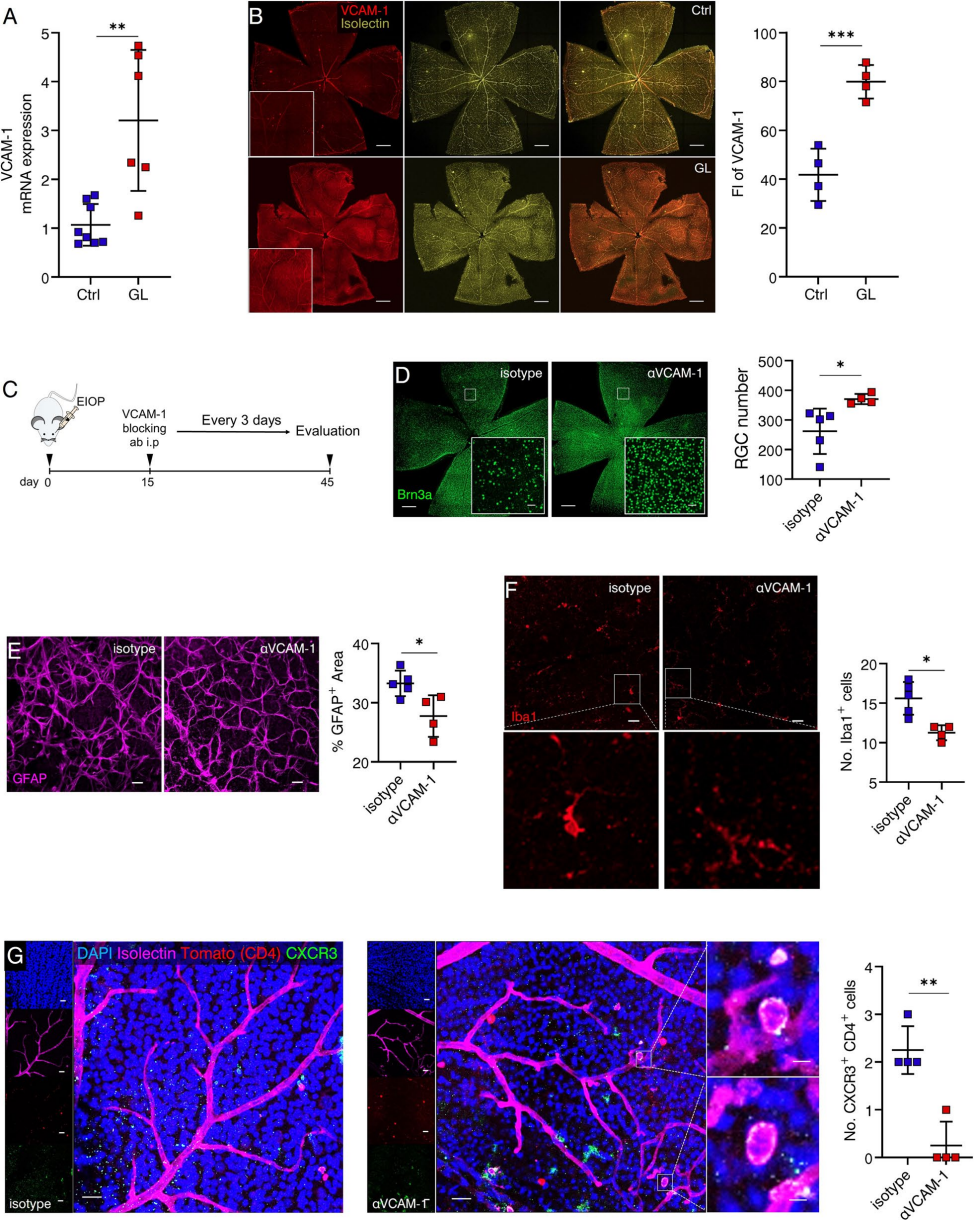
Retinal endothelial expression of VCAM-1 is critical for Th1 cell migration across the blood-retina barrier (BRB). **A** Shown are results representing one of three independent experiments. The mRNA expression of VCAM-1 in the retina of GL mice (day 20) was determined by qRT-PCR. Ctrl, *n* = 8; GL, *n* = 6. *n* refers to the number of RNA tests. Two retinas from 2 mice (one retina from each mouse) were combined for each RNA test. **B** Shown are results representing one of three independent experiments. Representative confocal images of retina flat-mounts stained for VCAM-1 from Ctr (*n* = 4) and GL (day 20, *n* = 4) (scale bar: 500 μm). Fluorescence intensity (FI) of VCAM-1 was quantified. *n* refers to the number of retinas used for staining and one retina was analyzed per mouse. **C** Experimental design: glaucoma was induced and mice were intraperitoneally injected with anti-VCAM-1 (αVCAM-1) or isotype antibody every 3 days from day 15 to the end of indicated experiments. **D**–**F** Shown are results representing one of three independent experiments. In each experiment, one random retina from each recipient mouse was used for RGC density and the other was for Iba1 and GFAP co-staining (isotype, *n* = 5; αVCAM-1, *n* = 4.* n* refers to the number of retinas). **D** RGC number, **E** the percentage of GFAP^+^ area per microscopic field (size: 319.45 μm^2^), and **F** the number of microglia (magnification: morphological changes) were quantified. **G** Shown are results representing one of three independent experiments. Immunofluorescence staining for Isolectin, CXCR3, and DAPI (blue) in retina flat-mounts of GL mice with CD4-specific expression of Tomato, treated with αVCAM-1 or isotype, and retinal CXCR3^+^ CD4^+^ T cells were quantified (isotype, *n* = 4; αVCAM-1, *n* = 4. *n* refers to the number of retinas used for staining and one random retina per mouse was used). **D** Scale bar: 500 μm; inset, 20 μm. **E**, **F** Scale bar: 20 μm. **g** Scale bar: 20 μm; inset, 5 μm. *P < 0.05, **P < 0.01, ***P < 0.001. two-tailed unpaired Student’s* t* test was performed

We have demonstrated the infiltration of Th1 cells into the glaucomatous retina and their significant contribution to RGC loss. This observation raises a fundamental question: how do circulating Th1 cells access the retina, considering the retina's immune-privileged status and the presence of BRB that normally separates circulating immune components from the neural retina? Cell adhesion molecules (CAMs) and chemokines play crucial roles in lymphocyte trafficking and immune responses within target tissues [33, 34]. In the CNS, VCAM-1 is one extensively studied adhesion molecule, which is involved in Th1 cell capture and arrest by the CNS endothelium [35], and implicated in T cell-mediated neuroinflammatory and neurodegenerative diseases [36]. Given the similarities between the retina and CNS in terms of their neural tissue nature and shared developmental and pathological mechanisms, we investigated the involvement of VCAM-1 in our glaucoma model. We observed a significant upregulation of VCAM-1 at the transcriptional level in glaucomatous retinas as early as day 20 following MB injection (Fig. 7A), matching the timing of Th1 cell infiltration into the retina (Fig. 5A). Using immunofluorescence staining of retina whole mounts, we confirmed the increased endothelial expression of VCAM-1 (Fig. 7B). To the best of our knowledge, this is the first report of VCAM-1 upregulation in retinal microvessels in glaucoma. To delve deeper into the role of VCAM-1, we performed intravitreal injection of anti-VCAM-1 antibody (Fig. 7C). The blockade of retinal VCAM-1 led to a significant amelioration of RGC loss (Fig. 6D), mitigated gliosis (Fig. 7E), diminished microglial activity (Fig. 7F), and importantly, reduced Th1 cell invasion (Fig. 7G). These findings underscore the contribution of VCAM-1 to neurodegeneration in the glaucomatous retina.

**Fig. 8:**
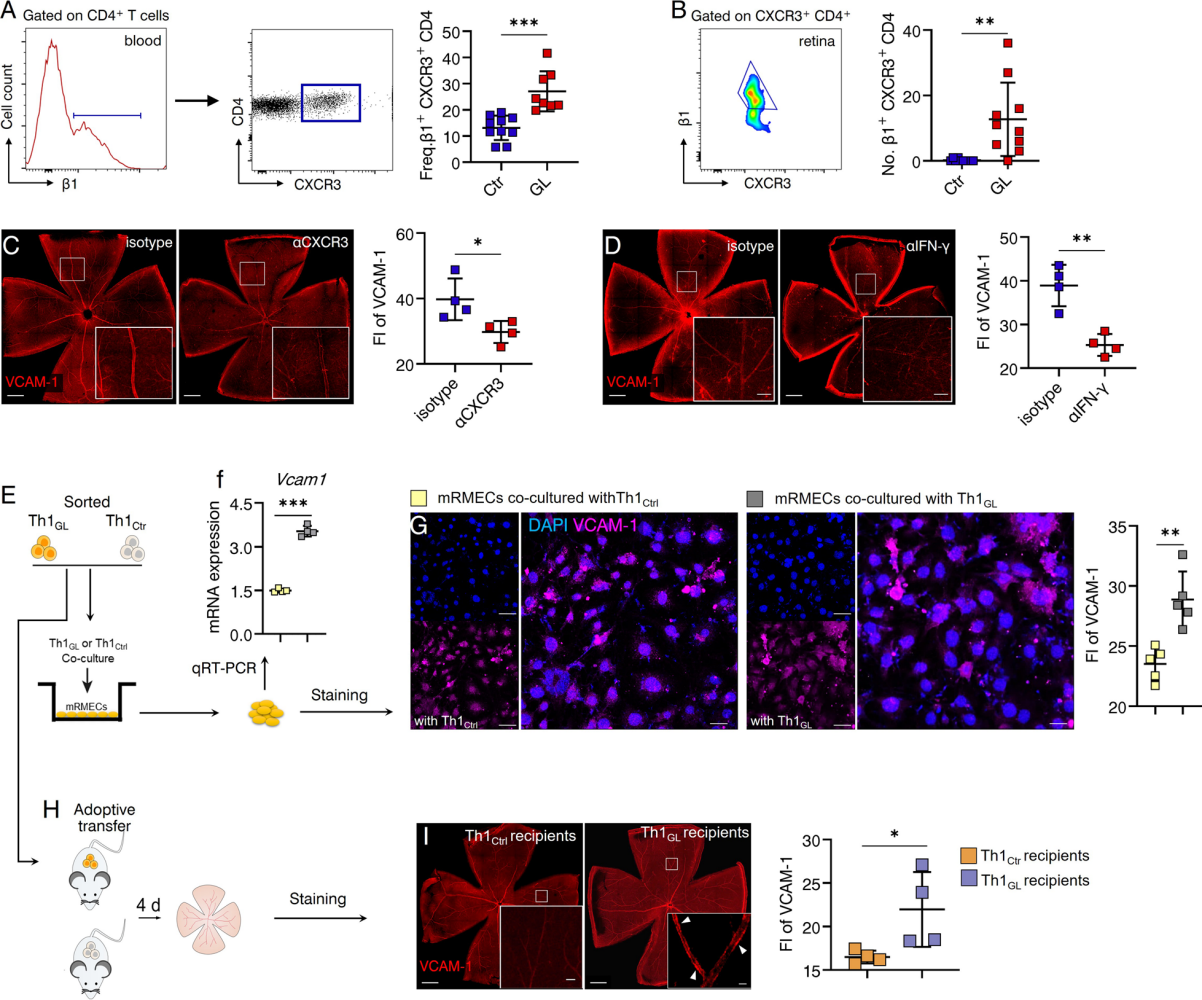
Th1 cells required for retinal endothelial VCAM-1 upregulation in glaucoma. **A** Flow cytometry was performed to quantify peripheral blood β1^+^ CXCR3^+^ CD4^+^ T cells (Ctr, *n* = 10; GL, *n* = 8) (*n* refers to the number of enrolled mice, and shown are representative results of one of three independent experiments). **B** Flow cytometry was performed to quantify retina-infiltrating β1^+^ CXCR3^+^ CD4^+^ T cells (Ctr, *n* = 10; GL, *n* = 10; shown are pooled data from multiple experiments; *n* refers to the number of flow cytometry tests; 4 retinas from 4 individual mice (one retina from each mouse) were combined for each flow cytometry test; the total number of enrolled mice were: Ctr = 40; GL = 40). **C**, **D** Shown are representative results of one of three independent experiment. **C** Anti-CXCR3 or **D** anti-IFN-γ treatment was performed as described in Fig. 5H or Fig. 4E. Retina flat-mounts were prepared and stained for VCAM-1 and fluorescence intensity (FI) of VCAM-1 was quantified. *n* = 4, *n* refers to the number of retinas used for staining and one random retina was analyzed per mouse. **E** Shown is a schematic of the cell co-culture design: Th1_GL_ (cells pooled from 8 mice) and Th1_Ctrl_ (cells pooled from 8 mice) cells sorted by FACS were co-cultured in vitro with mRMECs (a mouse retinal microvessel cell line), respectively. After 3 days of co-culture, each well was washed to remove non-adhesive T cells. **F** The mRNA expression of VCAM-1 in remaining cells was determined by qRT-PCR. Each RNA test for qRT-PCR was pooled from 3 wells of cells. *n* = 4 tests. **G** Immunofluorescence staining for VCAM-1 was performed after non-adhesive T cells were removed. Representative images of VCAM-1-expressing mRMECs (scale bar: left, 20 μm; right, 20 μm). VCAM-1 fluorescence intensity (FI) was evaluated. In each culture well, at least 3 microscopic fields (size: 159.73 mm^2^) were analyzed. *n* = 5. **H** Experimental design: Th1_GL_ and Th1_Ctr_ were labeled with a CellTrace CFSE Cell Proliferation Kit, and then transferred into naïve WT recipient mice via tail vein (2 × 10^6^ cells per recipient mice), respectively. After 4 days of reconstitution, all recipients were sacrificed and their retinas were collected. **I** Shown are results representing one of three independent experiment. Retina flat-mounts of Th1_GL_ and Th1_Ctr_ recipients were stained for VCAM-1 (scale bar: 500 μm; inset, 20 μm). For determination of VCAM-1 expression, fluorescence intensity (FI) was quantified. *n* = 4, *n* refers to the number of retinas used for staining for VCAM-1 and one random retina of each recipient mouse was analyzed. *P < 0.05, **P < 0.01, ***P < 0.001, two-tailed unpaired Student’s t test was performed

Subsequently, the expression of β1 integrin, the ligand for VCAM-1, was investigated in Th1 cells in the circulation and within the retina. Remarkably, both the circulation (Fig. 8A) and retina (Fig. 8B) exhibited increased levels of β1^+^ Th1 cells before the progressive loss of RGC, implying a potential causal relationship. To further explore this, two approaches were employed to interfere with Th1 cell functions. Both anti-CXCR3 (Fig. 8C) and anti-IFN-γ (Fig. 8D) interventions effectively ameliorated the upregulation of endothelial VCAM-1 in glaucomatous retinas. Furthermore, Th1_GL_ and Th1_Ctrl_ were sorted to for in vitro experiments (Fig. 8E). Co-culture of Th1_GL_ with mRMECs (a mouse retinal microvessel cell line) confirmed that Th1_GL_ cells were capable of inducing VCAM-1 expression in retinal endothelial cells (Fig. 8F, G). As postulated above, Th1 cells possess the ability to upregulate endothelial VCAM-1 expression, thereby facilitating their transmigration across the BRB. To further validate this supposition in an adoptive transfer model (Fig. 8H), we examined VCAM-1 expression in retinal microvessels of EIOP-naïve recipients after Th1_GL_ transfer, and indeed, an increased expression of VCAM-1 was observed (Fig. 8I). Collectively, these findings support the notion that Th1 cells contribute to RGC loss in glaucoma by promoting VCAM-1 expression in retinal microvessels, thus facilitating the transmigration of Th1 cells into the neural retina.

## Discussion

Glaucoma, a condition characterized by EIOP, poses several clinical challenges that have not been fully elucidated. While EIOP is recognized as the primary risk factor for glaucoma, emerging evidence supports the involvement of immune responses, including complement cascade activation and microglial activation, in glaucomatous degeneration. Both clinical and laboratory studies have demonstrated that immune responses occurring after the disease onset significantly contribute to the pathogenesis of glaucoma. T cells have been recently highlighted in glaucomatous RGC degeneration, as evidenced by transfer experiments from glaucomatous mice and studies on T cell-deficient mice [12, 18]. However, the mechanisms underlying the infiltration of circulating T cells across the BRB into the retina remain unclear. The current study presents three key findings that shed light on the pathogenesis of glaucoma by focusing on the crosstalk between T cells and retinas. These findings include: (i) Th1 cells undergo functional reprogramming in the early phase of glaucoma, enabling them to target the retina; (ii) infiltrated Th1 cells fostered a pro-inflammatory milieu through intricate interplay with retinal microglia, thus leading to RGC loss; (iii) retinal endothelial cells were affected by peripheral Th1 cells and upregulated VCAM-1 expression in glaucomatous mice, in turn facilitating Th1 cell invasion. These novel findings unveil a previously unknown mechanism through which peripheral T immunity regulates glaucomatous RGC loss, establishing a connection between the periphery immune system and the retina.

In neurodegenerative disorders like glaucoma, neuroinflammation constitutes a fundamental process wherein microglia play a pivotal role [4]. Microglia form the primary line of immune defense in both the retina and the CNS [37]. Following injury, these cells undergo activation including morphological changes, proliferation, migration towards the sites of damage, and modifications in the expression of enzymes and receptors. Excessive activation of microglia might induce the release of various inflammatory factors, leading to neuronal damage. The interaction between microglia and T cells has recently gained attention in the pathology of CNS diseases [31]. Upon injury, microglia can upregulate their expression of MHC II and CD86 to facilitate interaction with CD4^+^ T cells. A recent investigation has revealed that CD4^+^ T cells from patients with Parkinson's disease (PD) specifically respond to antigenic MHC class II epitopes derived from α-synuclein [38]. PD murine models lacking T cells, particularly CD4^+^ T cells, have demonstrated reduced cell death and microglial activation [39]. Moreover, Th1 cell-associated cytokines have been implicated in promoting microglial activation, leading to the acquisition of a phenotype that damages neurons in the CNS pathology [40–42]. Here, our study found evidence of Th1 cell infiltration into the retina before significant RGC loss occurred, indicative of a causal relationship between Th1 cell activity and glaucomatous RGC loss. Although the number of infiltrated Th1 cells was relatively small even in glaucomatous retinas, it has been suggested that sustained activity of even a small number of T cells can significantly reduce the survival of RGC. Infiltrated T cells can worsen microglial activation and lead to the release of damaging substances, thus establishing a pro-inflammatory environment that destroys RGCs through cell–cell communication [12, 43, 44]. To support this notion, our study found that: (i) microglia were observed to directly respond to Th1_GL_ cells in an in vitro co-culture, (ii) transfer of Th1_GL_ cells into mice with no prior exposure to EIOP resulted in the presence of Th1_GL_ cells and overactivated microglia in the recipient retinas, and (iii) systemic blockade of Th1 cells effectively attenuated RGC loss and detrimental microglial activities. Consequently, our findings suggest that targeting circulating Th1 cells may hold promise as a potential therapeutic strategy to mitigate progressive glaucomatous neural damage, particularly in patients with controlled IOP. Further research in this area may unveil innovative treatments for glaucoma.

Circulating T cells must traverse the BRB and exit the retinal microvessels to reach the neural retina. This process, known as "lymphocyte transendothelial migration", is tightly regulated by a series of intricate events involving selectin-ligand interactions, chemokines, and CAMs [45, 46]. Among these CAMs, VCAM-1 stands out as a crucial adhesion molecule expressed on activated endothelial cells. Once expressed on the endothelial cell surface, VCAM-1 binds to its ligands on intravascular leukocytes, particularly the α4β1 integrin (also known as VLA-4 or CD49d/CD29) expressed on leukocytes, thereby facilitating their transendothelial migration. Although the role of VCAM-1 has been studied in various diseases, such as multiple sclerosis, a neuroinflammatory condition affecting the central nervous system [47], there is limited information concerning its involvement in retinal diseases. However, recent studies have implicated VCAM-1 as a major driver of neovascularization in diabetic retinopathy [48]. Hyperglycemia and hyperlipidemia have also been shown to induce VCAM-1 expression in retinal vessels of mice. Despite these findings, the role of VCAM-1 in glaucoma remains unknown. Therefore, our study aimed to investigate the involvement of VCAM-1 in glaucoma pathogenesis. Our data demonstrated a significant upregulation of retinal endothelial VCAM-1 in glaucomatous mice, which facilitated the transmigration of circulating Th1 cells across the BRB. Remarkably, blocking retinal VCAM-1 attenuated Th1 cell invasion and mitigated progressive glaucomatous RGC damage. Moreover, although normal retinal endothelial cells express VCAM-1 at low levels, the mechanisms underlying its upregulation in glaucomatous retinas remain elusive. Previous studies have shown that Th1-specific cytokine IFN-γ is a potent inducer of VCAM-1 expression on brain endothelial cells, promoting the transmigration of Th1 cells across the blood–brain barrier [49]. Our findings support this hypothesis: (i) systemic blockade of Th1 cells or IFN-γ significantly attenuated retinal endothelial VCAM-1 upregulation in glaucomatous retinas; (ii) adoptive transfer of Th1_GL_ cells into mice without EIOP stress resulted in VCAM-1 upregulation in the retinal microvessels of recipients; (iii) in vitro coculture experiments further confirmed the upregulation of VCAM-1 on retinal endothelial cells by Th1_GL_ cells. Therefore, it is plausible that peripheral inflammation, such as the activation of Th1 cells, can trigger an inflammatory cascade in the retina, compromising the BRB's integrity. This, in turn, allows the entry of peripheral inflammatory components, contributing to a positive feedback loop of inflammation. Collectively, these results shed light on the role of endothelial VCAM-1 in glaucoma pathogenesis and suggest that enhancing retinal microvessel expression of VCAM-1 might be one mechanism by which circulating Th1 cells breach the BRB and invade the retina.

The increased expression of VCAM-1 represents a downstream effect of pro-inflammatory cytokines, facilitating leukocyte recruitment to the site of inflammation. Blocking the VCAM-1-ligand interaction has emerged as a successful therapeutic approach for autoimmune diseases [50]. Glaucoma, an incurable and irreversible neurodegenerative condition that leads to blindness, lacks sufficient treatment options. The reduction of IOP is currently the only proven method to slow down the progression of glaucoma. However, the effectiveness of IOP-lowering treatments in providing neuroprotection is sometimes limited. If peripheral and retinal immune events, similar to those described in our current study, occur in glaucoma patients, it becomes crucial to develop innovative therapies targeting specific immune components for the prevention of continued or recurrent episodes of vision loss, even in the absence of EIOP. In the current study, animal experiments demonstrated the critical role of the interaction between β1^+^ Th1 cells and VCAM-1^+^ endothelial cells in glaucoma pathogenesis, and therapeutic strategies targeting this interaction were effective. Although there are currently no clinically approved agents targeting VCAM-1, Natalizumab, an agent that targets α4β1 and effectively blocks VCAM-1-α4β1 interaction, has become one of the most effective therapies for multiple sclerosis [51]. Moreover, our data showed that intravitreal injection of anti-VCAM-1 antibody effectively blocked the influx of circulating Th1 cells and alleviated retinal neurodegeneration, making it an attractive candidate for therapeutic intervention in glaucoma.

Another significant finding in our study is the overactivation of peripheral CD4^+^ T cells in both human and experimental glaucoma, and developed Th1-biased responses. The activation status of CD4^+^ T cells can be discerned by the distribution of their naïve, central memory, and effector memory subsets within the peripheral blood [52]. Upon exposure to antigenic activation signals, CD4^+^ T cells become activated and exhibit effector functions. However, the majority of these cells ultimately undergo cell death. The surviving cells are long-lived cells, mainly consisting of central memory (Tcm) and effector memory (Tem) subsets. Unlike Tcm, Tem cells lack expression of CCR7, a crucial molecule for lymph node homing and T cell compartmentalization within lymph nodes. Consequently, Tem cells display a predilection for migration to non-lymphoid sites, such as inflamed tissues, rendering them "primed for action". In contrast, Tcm cells resemble naïve T cells in that they express CCR7 and are more inclined to traffic to lymph nodes, affording them a "quiescent" state [53, 54]. However, upon re-encountering the antigen, Tcm cells can rapidly downregulate CCR7 expression and regain effector functions. Our findings demonstrate that the activation of circulating CD4^+^ T cells precedes significant RGC loss, and their depletion significantly reduces glaucomatous retinal neurodegeneration, indicating a causative link. Moreover, circulating CD4^+^ T cell migration can be targeted by clinically approved drugs such as Fingolimod, an oral sphingosine-1-phosphate receptor modulator that modulates immune responses and reduces CD4^+^ T cell infiltration into inflammatory sites such as the brain, by selectively sequestering lymphocytes within secondary lymphoid organs [55]. This unique mechanism of action establishes Fingolimod as a potent immunomodulatory agent with promising applications in various neurological disorders, including multiple sclerosis [56]. Based on these findings, it is reasonable to expect future clinical studies using drugs like Fingolimod for glaucoma treatment.

Despite the findings in our study, several limitations should be acknowledged. (i) The mechanisms by which EIOP affects circulating CD4^+^ T cell biology and function in the early phase of glaucoma remain largely unknown. This was mainly due to the lack of existing research in this specific area, leaving us with limited references to draw upon. The current gaps need further investigation in order to fully comprehend the intricate relationship between EIOP stress and T cell dysfunction. (ii) In the current study, we did not perfuse the mice when subjected to retinal immune profile analysis. It is worth noting that leukocyte transendothelial migration comprises several intricate stages, such as adhesion and rolling on endothelial cells, which play a crucial role in leukocyte extravasation [32, 57]. Furthermore, the presence of retinal leukocyte stasis has been observed in retinal pathology and is believed to contribute to the pathogenesis of the disease. Considering these factors, in our present study, we did not conduct retinal microvascular perfusion to selectively eliminate immune cells from the vasculature before harvesting the retina. However, to further elucidate and differentiate the potential differences and nature of immune cells within and outside the retinal vasculature, microvascular perfusion is required in future studies. (iii) The interactions between leucocytes and endothelial cells, followed by the transendothelial migration, play a critical role in enabling immune cells to breach the BRB. As leucocyte transendothelial migration involves a series of complex steps, it is likely that different adhesion and activation molecules are involved in securing various stages of this extravasation cascade. While our study highlights the involvement of VCAM-1 in the recruitment of circulating Th1 cells into the retina, it is important to note that there are likely other molecules that potentially contribute to this process. However, the specific roles of these molecules remain largely uncertain and require further investigation. (iv) The interaction between T cells and retinal components (eg, microglia, neurons) is critical for the local environment that damages RGC. Our study indicates the involvement of Th1 cells in the modulation of retinal glial activity, but the underlying cellular and molecular mechanisms require further elucidation. Addressing these gaps in knowledge will require a series of follow-up studies in the future.

Our study provides novel evidences showing the invasion of circulating Th1 cells into the retina through VCAM-1-mediated transendothelial migration in glaucoma, subsequently leading to retinal neurodegeneration. To our best knowledge, this study is the first to shed light on this previously unexplored aspect. These findings highlight the crucial role of peripheral immunity in the pathogenesis of glaucoma, emphasizing the need to consider immune-mediated mechanisms in understanding and treating glaucoma. Moreover, our study opens up new avenues for the development of innovative therapeutic interventions that target immune pathways in glaucoma management.

### Supplementary Information

**Additional file 1: Fig. S1.** Association of circulating CD4^+^ T cell response with the cup-to-disc ratio in patients with glaucoma. CD4^+^ T cell status was determined as described in Fig. 1. Shown are comparison of Freq. EM CD4^+^, CM CD4^+^, and EM/CM among different groups stratified according to cup-to-disc ratio (C/D) enlargement. Statistical comparisons were performed using Kruskal-Wallis test followed by Dunn's multiple comparisons test. **Fig. S2.** The depletion of CD4^+^ T cells reduce retinal GFAP expression in glaucoma. The depletion of CD4+ T cells was performed as described in Fig. 3L. The percentage of GFAP^+^ area per microscopic field (size: 319.45 μm^2^) was calculated.* n* = 4, *n* refers to the number of retinas used for GFAP staining. Only one retina per mouse was used. Results presented are representative of three independent experiments. ***P < 0.001, two-tailed unpaired Student’s* t* test was performed. **Fig. S3.** CXCR3 expression on IFN-γ-producing CD4^+^ T cells. Murine CD4^+^ T cells (n = 8) were stimulated with PMA and ionomycin in the presence of GolgiStop. Flow cytometry was performed to determine CXCR3 and IFN-γ expression. The table is showing individual data of the percentage of the CXCR3^+^ population in total IFN-γ-producing CD4^+^ T cells. **Table S1.** Demographics of glaucoma patients and healthy controls. **Table S2.** Antibodies used for immunofluorescent staining.

## Data Availability

The data underlying the research results are available in the article.
