# Supplementary material for: Th1 cells contribute to retinal ganglion cell loss in glaucoma in a VCAM-1-dependent manner
Source: J Neuroinflammation. 2024 Feb 5;21:43. doi: 10.1186/s12974-024-03035-5 (PMC10840227; doi:10.1186/s12974-024-03035-5)
Supplement: Supplementary file 1 — Additional file 1: Fig. S1. Association of circulating CD4+ T cell response with the cup-to-disc ratio in patients with glaucoma. CD4+ T cell status was determined as described in Fig. 1. Shown are comparison of Freq. EM CD4+, CM CD4+, and EM/CM among different groups stratified according to cup-to-disc ratio (C/D) enlargement. Statistical comparisons were performed using Kruskal-Wallis test followed by Dunn's multiple comparisons test. Fig. S2. The depletion of CD4+ T cells reduce retinal GFAP expression in glaucoma. The depletion of CD4+ T cells was performed as described in Fig. 3L. The percentage of GFAP+ area per microscopic field (size: 319.45 μm2) was calculated. n = 4, n refers to the number of retinas used for GFAP staining. Only one retina per mouse was used. Results presented are representative of three independent experiments. ***P < 0.001, two-tailed unpaired Student’s t test was performed. Fig. S3. CXCR3 expression on IFN-γ-producing CD4+ T cells. Murine CD4+ T cells (n = 8) were stimulated with PMA and ionomycin in the presence of GolgiStop. Flow cytometry was performed to determine CXCR3 and IFN-γ expression. The table is showing individual data of the percentage of the CXCR3+ population in total IFN-γ-producing CD4+ T cells. Table S1. Demographics of glaucoma patients and healthy controls. Table S2. Antibodies used for immunofluorescent staining. [file 12974_2024_3035_MOESM1_ESM.docx]

**Additional Figures and Figure Legends**

**
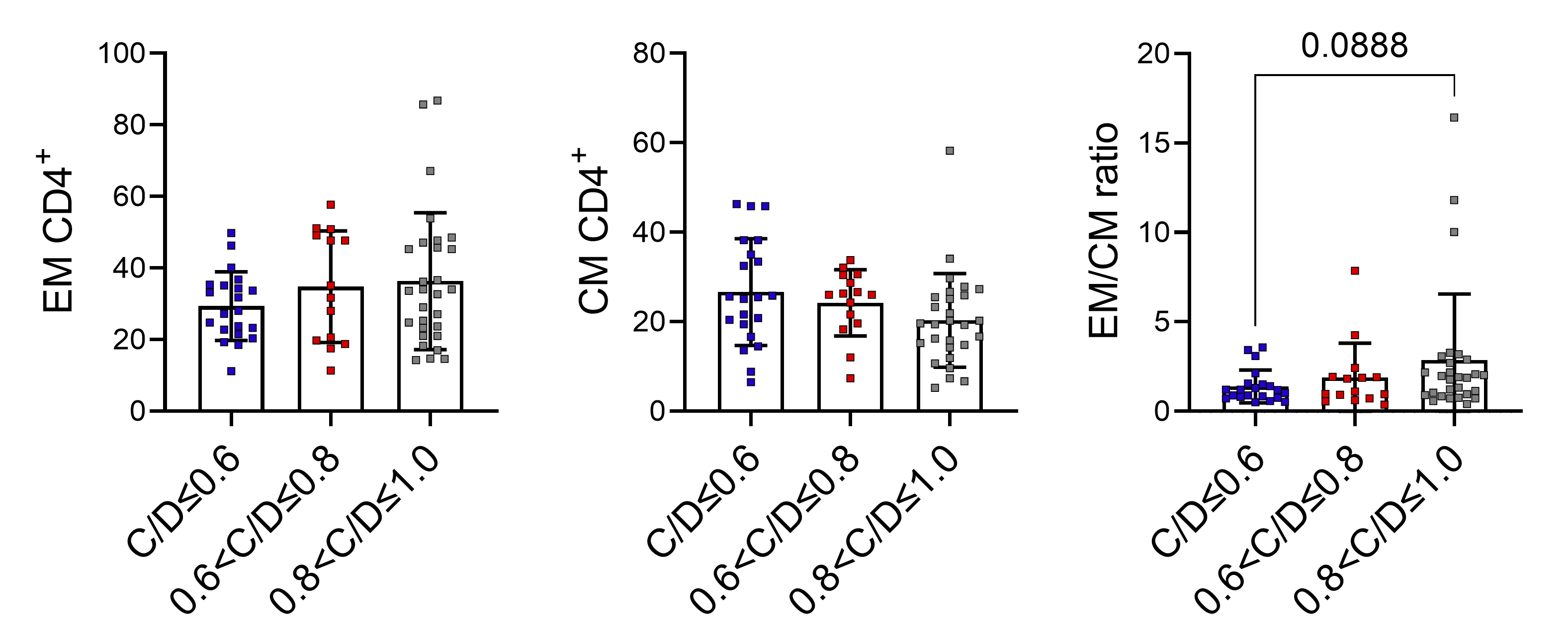
**

Fig. S1 Association of circulating CD4^+^ T cell response with the cup-to-disc ratio in patients with glaucoma. CD4^+^ T cell status was determined as described in **Fig. 1**. Shown are comparison of Freq. EM CD4^+^, CM CD4^+^, and EM/CM among different groups stratified according to cup-to-disc ratio (C/D) enlargement. Statistical comparisons were performed using Kruskal-Wallis test followed by Dunn's multiple comparisons test.

**
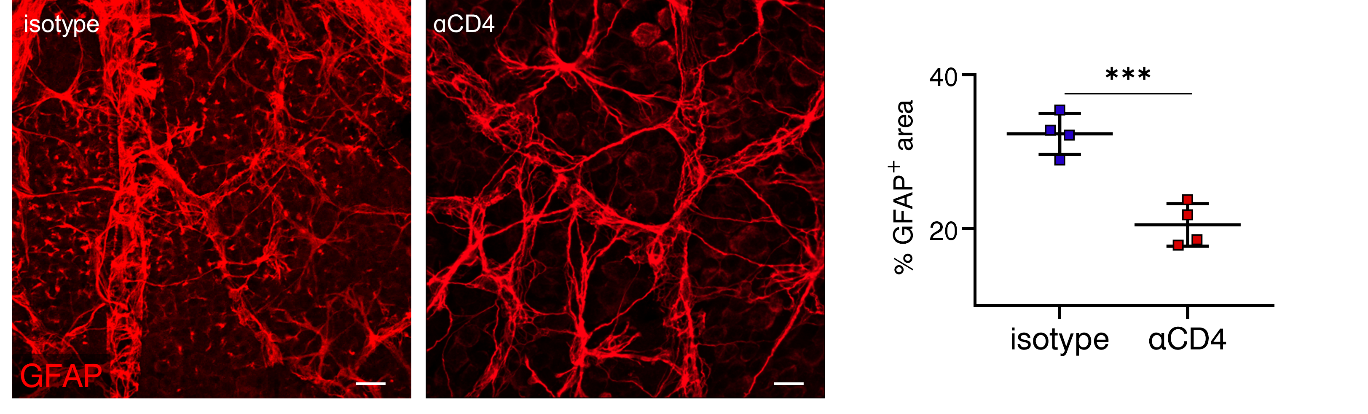
**

Fig. S2 The depletion of CD4^+^ T cells reduce retinal GFAP expression in glaucoma. The depletion of CD4+ T cells was performed as described in Fig. 3L. The percentage of GFAP^+^ area per microscopic field (size: 319.45 μm^2^) was calculated. *n* = 4, *n* refers to the number of retinas used for GFAP staining. Only one retina per mouse was used. Results presented are representative of three independent experiments. ****P* < 0.001, two-tailed unpaired Student’s *t* test was performed.

**
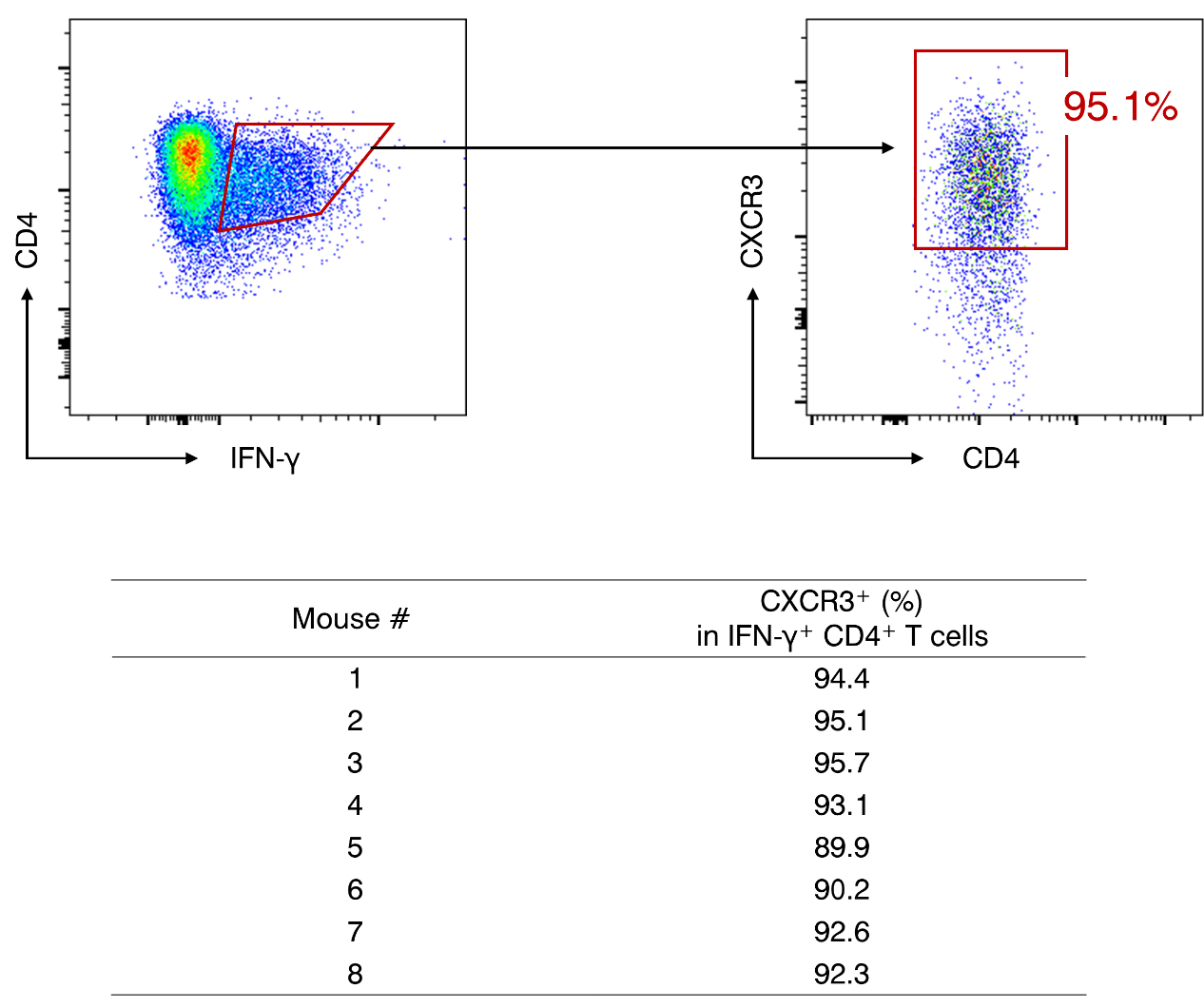
**

Fig. S3 CXCR3 expression on IFN-γ-producing CD4^+^ T cells. Murine CD4^+^ T cells (n = 8) were stimulated with PMA and ionomycin in the presence of GolgiStop. Flow cytometry was performed to determine CXCR3 and IFN-γ expression. The table is showing individual data of the percentage of the CXCR3^+^ population in total IFN-γ-producing CD4^+^ T cells.

**Additional Tables**

**Table. S1| Demographics of glaucoma patients and healthy controls**

|  | GL  (Cohort #1) | HC  (Cohort #1) | *p* value | GL  (Cohort #2) | HC  (Cohort #2) | *p* value |
| --- | --- | --- | --- | --- | --- | --- |
| n | 87 | 98 |  | 113 | 121 |  |
| Age (year) * | 59 (49−68) | 56 (42−65) | 0.07 | 60 (52−68) | 55 (41-67) | 0.14 |
| Gender |  |  |  |  |  |  |
| Female | 48 | 54 | 0.99 | 63 | 76 | 0.27 |
| Male | 39 | 44 |  | 50 | 45 |  |
| PACG/POAG | 60/27 | - |  | 82/31 | - |  |
| IOP (mmHg) * | 21.1 (14.9−29.2) | 15.3 (13.5−19.0) | < 0.001 | 23.7 (19.4−38.2) | 16.2 (14.4-17.9) | < 0.001 |
| Severity |  |  |  |  |  |  |
| Mild | 28 | - |  | 33 | - |  |
| Moderate | 10 | - |  | 14 | - |  |
| Severe | 49 | - |  | 66 | - |  |

* Data are presented as median (IQR).

Abbreviations: GL, glaucoma; HC, healthy controls; IQR, interquartile range; PACG, primary angle-closure glaucoma; POAG, primary open-angle glaucoma; IOP, intraocular pressure.

Kolmogorov-Smirnov test was performed for checking data normality. The differences of all parameters between glaucoma patients and healthy controls were examined by Mann-Whitney test, except for the gender, which was examined by Chi-square test. *p* < 0.05 was considered statistically significant.

**Table. S2| Antibodies used for immunofluorescent staining**

| **Primary Antibodies** | **Source** | **Clone** | **Corresponding Isotype Control** | **Dilution Ratio** |
| --- | --- | --- | --- | --- |
| Brn3a | Abcam | EPR23257-285 | Rabbit IgG, monoclonal (Abcam, clone EPR25A) | 1:500 |
| GFAP | Neuromics | - | Rabbit IgG, monoclonal (Abcam, clone EPR25A) | 1:500 |
| SMI32 | Biolegend | SMI32 | [Mouse IgG1 monoclonal (Abcam, clone 1R312)](https://www.abcam.cn/products/primary-antibodies/mouse-igg1-monoclonal-r312-mouseigg1-isotype-control-bsa-and-azide-free-ab281291.html) | 1:500 |
| SMI34 | Biolegend | SMI34 | Mouse IgG1, kappa monoclonal (Abcam, clone 15-6E10A7) | 1:500 |
| Iba1 | Wako | - | Rabbit IgG, monoclonal (Abcam, clone EPR25A) | 1:200 |
| CD4 | Abcam | EPR19514 | Rabbit IgG, monoclonal (Abcam, clone EPR25A) | 1:200 |
| CXCR3 | Boster | - | Rabbit IgG, monoclonal (Abcam, clone EPR25A) | 1:200 |
| VCAM-1 | CST | D8U5V | Rabbit IgG, monoclonal (Abcam, clone EPR25A) | 1:100 |
| Isolectin GS-IB4 | Thermo Fisher Scientific | - | Mouse IgG1, kappa monoclonal (Abcam, clone 15-6E10A7) | 1:200 |
| DAPI | Invitrogen | - | - | 1:10000 |
| CXCL10 | RD | - | [Goat IgG, polyclonal (Abcam)](https://www.abcam.cn/products/primary-antibodies/goat-igg-polyclonal-isotype-control-ab37373.html) | 1:10 |
| **Second Antibodies** | **Source** | **Clone** | - |  |
| Goat anti-Mouse IgG (H+L) Highly Cross-Adsorbed Secondary Antibody, Alexa Fluor Plus 488 | Thermo Fisher Scientific | _ | _ | 1:800 |
| Goat anti-Mouse IgG (H+L) Highly Cross-Adsorbed Secondary Antibody, Alexa Fluor Plus 555 | Thermo Fisher Scientific | _ | _ | 1:800 |
| Goat anti-Rabbit IgG (H+L) Highly Cross-Adsorbed Secondary Antibody, Alexa Fluor Plus 488 | Thermo Fisher Scientific | _ | _ | 1:800 |
| Goat anti-Rabbit IgG (H+L) Highly Cross-Adsorbed Secondary Antibody, Alexa Fluor Plus 555 | Thermo Fisher Scientific | _ | _ | 1:800 |
| Goat anti-Rabbit IgG (H+L) Highly Cross-Adsorbed Secondary Antibody, Alexa Fluor Plus 647 | Thermo Fisher Scientific | _ | _ | 1:800 |
| Goat anti-Rat IgG (H+L) Cross-Adsorbed Secondary Antibody, Alexa Fluor 594 | Thermo Fisher Scientific | _ | _ | 1:500 |
